# Interaction between official institutions and influential users of rumor control in online social networks

**DOI:** 10.3389/fpsyg.2022.937296

**Published:** 2022-08-02

**Authors:** Shizhen Bai, Wenya Wu, Man Jiang

**Affiliations:** School of Management, Harbin University of Commerce, Harbin, China

**Keywords:** game theory, COVID-19, rumor clarification, rumor verification, online social networks

## Abstract

Online interactions have become major channels for people to obtain and disseminate information during the new normal of COVID-19, which can also be a primary platform for rumor propagation. There are many complex psychological reasons for spreading rumors, but previous studies have not fully analyzed this problem from the perspective of the interaction between official institutions and influential users. The purpose of this study is to determine optimal strategies for official institutions considering the impact of two different influential user types (trolls and reputed personalities) by designing two game-theoretic models, namely “Rumor Clarification and Interaction Model” and “Rumor Verification and Interaction Model,” which can, respectively decide whether to clarify and when to clarify. The results of this article show that clarification strategies can be decided according to the characteristics of rumors and the influential user’s reactions. Meanwhile, publishing verified information prevents trolls’ “loophole advantages” and prevents reputed personalities from spreading false information due to the vague authenticity of rumors. Results also show that the verification strategy is limited by cost, period, and verification index.

## Introduction

During the new normal brought by the COVID-19 pandemic, online interaction has become a primary method of connecting people and disseminating information globally. Although Online Social Networks (OSNs) provide a great convenience for people to interact andoften serve as a platform for countries to release critical decisions during disastrous events (Ngamassi et al., 2016; Subramaniyaswamy et al., 2017), it also speeds up the spread of misinformation, such as rumors, in an unprecedented short time (Islam et al., 2020), and thus threaten public order and social stability (McKee et al., 2019). How to clarify and control online rumors has attracted significant attention from the research community (Hosni et al., 2020b; Wang X. et al., 2021; Zareie and Sakellariou, 2021; Agarwal et al., 2022; Yao et al., 2022).

In literature, the most common strategies to control rumors can be classified into three categories: (1) Spreading truth to clarify rumors (Weeks and Garrett, 2014; Pal et al., 2020; Yao et al., 2021); (2) Verifying the authenticity of information (Flanagin and Metzger, 2007; Lee and Sundar, 2013; Chua and Banerjee, 2018); (3) Blocking influential users (Chen et al., 2019; Yan et al., 2019). Previous studies have provided useful guidelines for clarifying and controlling rumors, which laid a solid foundation for making the rumor control strategy. To the best of our knowledge, no previous study has considered the strategic interactions between official institutions and influential users during the rumor verification and clarification process.

Rumors in OSNs can be defined as stories or statements that have not been authentically verified or refuted by the authorities during their spreading in the network (DiFonzo and Bordia, 2007), which can be classified as exaggerations, fabrications, explanations, and astrological predictions (Prasad, 1935). It is generally believed that rumors arise in crucial, uncertain, potentially threat-causing, uncontrollable, and public panic circumstances (DiFonzo, 2008). The general public typically lacks theoretical knowledge and critical thinking, and words and choices provided by those influential users around them will influence their decision-making, resulting in conformity psychology (Zhang et al., 2022). Novel coronavirus pneumonia provides a new background, material, and driving mechanism for the online propagation of false information and rumors (Rovetta and Bhagavathuala, 2020). For example, even if someone searches for information about how to prevent new coronavirus virus infection, they can obtain many different answers, such as gargling with brackish salt, chewing garlic, taking antibiotics, smoking, and drinking. Therefore, masks, alcohol, and drugs were snapped up due to unclear information and panic psychology at the beginning of 2020. With the launch of epidemic prevention and universal vaccination programs, and with the rumor clarification by official institutions, news media, and some opinion leaders, such as Zhong Nanshan, an academician, people gradually understand the new coronavirus (Ruan et al., 2019). However, the epidemic continues to occur all over the world, and a variety of new rumors spread widely on social networks. Compared with the public health crisis caused by the epidemic, the massive dissemination of false information will cause a crisis of trust and even put public health at risk.

Over the past few years, numerous studies have characterized the propagation, detection, and control of rumors in OSNs (Ahsan et al., 2019; Askarizadeh and Ladani, 2021; Zareie and Sakellariou, 2021). Specifically, Vosoughi et al. (2018) found that compared with real news, false rumors have the characteristics of more novelty, faster propagation speed, and greater influence. Therefore, people are more likely to believe and spread rumors compared with real news (Wang and Zhuang, 2018). In the past decade, research on automatic algorithms, such as natural language processing, data mining, and machine learning, has made rumor detection more accurate (Wang and Guo, 2020; Parimi and Rout, 2021; Rani et al., 2022). In addition, many studies have protected online social networks from rumors, such as understanding and debunking rumors using a content analytic method (Song et al., 2021) and “anti-rumor” information propagation as a protection mechanism (Askarizadeh et al., 2019; Xiao et al., 2019).

As shown in the review above, previous studies have primarily focused on the propagation dynamics of rumors on social networks and people’s actions in the face of rumors. In reality, many rumors have been detected and blocked before they were spread, but many also spread to social networks. The most common means to control rumors is to release clarification information by official agencies and media companies. Due to limited resources, official rumor-refuting agencies must make strategic clarification strategies for various rumors while considering the potential tradeoffs between the cost of clarifying rumors and the impact of rumors on online social networks (Agarwal et al., 2022). There can be some unofficial individual users in a social network who have numerous followers, and their comments can be more influential than others. These users play a dual role in both spreading and controlling rumors on social networks (Ma et al., 2019). It is helpful for official agencies to control rumors if they release correct information. In contrast, this process will affect the cognition of their followers and thus aggravate the panic caused by rumors if they spread false information. Unfortunately, some interest groups also have strong effects. To make a profit, these groups may only want to spread rumors regardless of the truth, which has a direct impact on people’s understanding and judgment of those rumors (Lingam et al., 2018). Therefore, it is important to study the interaction between the rumor control strategy of official institutions and the behavior of influential users in social networks in the face of rumors.

Motivated by the fact that few scholars have investigated the strategic interaction between official institutions and influencers in social media during rumor clarification and verification, and the direction for future research proposed by Agarwal et al. (2022), this article completes the following work:

(a) This article creatively discusses the strategic interactions between official rumor control institutions and two types of influential users (trolls and reputed personalities) during the rumor clarification and verification.
(b) This article designs two game-theoretic models that consider the interaction behavior between official agencies and influential users (trolls and reputed personalities) to minimize the cost of rumor clarification and the influence of the rumor in an online social network.
(c) The first model, “Rumor Clarification and Interaction Model,” acts as a decision-making tool for the official rumor control agencies to make critical strategies on whether to clarify rumors by considering the cost and the impact of rumors and the choice that social users make due to the decisions of official institutions.
(d) The second model, “Rumor Verification and Interaction Model,” can be used to determine the best strategy for rumor control institutions to verify the information and address the issue that the trolls’ “loophole advantage” and reputed personalities make the wrong choices due to unclear rumor information.

The remainder of this article is organized as follows. Section “Related works” reviews related research on rumor clarification, rumor verification, and the role of influential users in social networks. Section “Rumor Clarification and Interaction Model” presents the Rumor Clarification Interaction Model and then proposes some insights based on numerical simulation results of the model. Section “Rumor VERTIFICATION and Interaction Model” describes the Rumor Verification Interaction Model and then provides some analysis and suggestions that are derived from the numerical results of the model. Section “Conclusion” concludes the article, and the “Supplementary Appendix” proves the proposition mentioned in this article.

## Related works

Internet rumor propagation disrupts the normal social communication order of society and impacts the social trust system to some extent. Studying the rumor propagation mechanism and controlling rumors have attracted the attention of both researchers and practitioners in recent years. This section presents a brief overview of the related works on rumor propagation and control strategies in OSNs.

### Rumor clarification

Government agencies and social media companies have the obligation to resist rumors, prevent falsehoods from spreading, and spread the truth. An important way to curb rumors in OSNs is to release clarification information by official institutions (Wen et al., 2014). During COVID-19, the WHO, the Federal Emergency Management Agency (FEMA), and China’s government created a “COVID-19” column on their website to report the latest situation and clarify rumors. For example, the Cyberspace Administration of China (CAC) has recommended several fact-checking tools to help the public identify and report rumors, such as the “Truth Check” platform of Tencent News (Cyberspace Administration of China, 2020). As China’s largest online microblog platform, Sina Weibo is a major platform for rumor spreading and has been a major rumor clarification platform during COVID-19. According to the official statistics in a report released by Sina Weibo Piyao (2020), the daily reported amount of false information was between 2,000 and 4,500 in the statistical range of September 2021, among which 5,512 rumors were effectively handled before the public could see them and 97 pieces of rumor clarification information were released to microblogs.

To effectively prevent the spread of online rumors and to reduce their negative effects, official authorities and social media primarily control rumors by using two external coercive strategies: controlling influential users to spread rumors and publishing rumor clarification information (Wen et al., 2014; Pal et al., 2020). The study found that publishing clarification information is more effective than blocking rumors in the long run because the openness of the internet makes it difficult to limit rumor spreading: the more a rumor is blocked from spreading, the easier it is to arouse people’s curiosity and skepticism. Therefore, the primary method of rumor control is increasingly inclined to be rumor clarification (Weeks and Garrett, 2014). Yang et al. (2020) proved that seeding correct information in the proximity of rumor seeds can minimize rumor spread in social networks with a heuristic algorithm based on diffusion dynamics. Official agencies can also select and use trusted users to disseminate clarification information, but these strategies often required high costs and time, which limits the number of effective clarification rumors (Wang et al., 2019). Srinivasan and Ld, 2021a focused on a collective rumor containment approach to control the rumor by spreading the correct information. Some scholars have considered effectiveness, for example, Li et al. (2021) identified key factors influencing rumor refutation effectiveness index when spreading truths to clarify the rumors. Some scholars have considered cost, for example, Yao et al. (2021) focused on how to find the social users with the least reputation to clarify rumors within a given time. Given the limited resources and the high cost of publicly disclosing online rumors, this article believes that official institutions and media must strategically choose rumor clarification strategies to improve efficiency and minimize the impact of rumors on social networks.

### Rumor verification

Clarifying rumors with unverified information may leave room for speculation and lead to serious harmful effects. For example, people with ulterior motives may catch loopholes and spread rumors for profit (Lingam et al., 2018), and some reputed personalities may release false information to their followers due to uncertainty (Pfeffer et al., 2014), thus accelerating the spread of rumors. In the early days of COVID-19, Li Wenliang, an ophthalmologist at Wuhan Central Hospital, first released epidemic warning information. However, his remarks were regarded as “creating panic” and “untrue remarks” by Wuhan policy at that time. This situation has also made the Chinese government strongly criticize the international community for taking containment measures without fully verifying information (Ashley Collman, 2020). Such incidents confirm the necessity for official agencies and social media companies to clarify rumors with verified information.

It is believed that official agencies and social media companies have the responsibility and resources to perform strict verification procedures before publishing information (Flanagin and Metzger, 2007). To verify rumor information, a variety of different approaches can be adopted, such as checking the primary and supporting sources. In general, compared with rumors, real information is more likely to be hyperlinked to trusted sources (Chua and Banerjee, 2018). Also, formal or trusted sources promote the dissemination of real information on social media (Lee and Sundar, 2013). Considering the limited time and resources of official institutions or news media and the different characteristics of each rumor, this article makes a decision between rapid clarification and spending energy on information verification.

### Influential users

Due to the scale-free nature of social networks (Zhuang et al., 2017), the propagation of rumors in OSNs depends on a specific group of users, called influencers (Zhuang et al., 2021). It is found that the number of rumors retweets and clarification information retweets are positively correlated with the number of fans (Chua et al., 2017) because users tend to trust the message published by someone they follow (Margolin et al., 2018) and influential people often have many followers. For example, domain experts or reputed personalities in social media typically receive more replies than ordinary users (Yang et al., 2018). Zubiaga et al. (2016) analyzed a rumor dataset to understand how users support, oppose or neutrally participate in rumor spreading and to explore the role of different types of users in the rumor propagation and clarification process. Therefore, identifying influential users in online social networks is an important study to accelerate the spread of information or block the spread of harmful content like rumors (Al-Garadi et al., 2018).

Scholars have different views on the role of influential users in the process of rumor dissemination and clarification. Some scholars believe that identifying and isolating those influential users helps to block rumor spreading (Chen et al., 2019; Yan et al., 2019), and others believe that influential communicators in social networks can be found to publish anti-rumor information (Srinivasan and Ld, 2021b). In terms of considering the double-edged sword impact of influential users, Hosni et al. (2020a) believe that some users may publish information wantonly regardless of its reliability to make profits through the inherent mechanism of OSNs. In addition, He et al. (2016) considered two cost-effective strategies, combined with the regular dissemination of truth and preventing influential users from participating in rumor dissemination to suppress rumors. Considering the positive effects of reputed personalities, Wang Y. et al. (2021) verified the key role of social media practitioners and opinion leaders in the spread and control of rumors and proposed some suggestions for official agencies to resist rumors from the perspective of considering social users. The perspective in this article is that influential people cannot be simply divided into opinion leaders or profit seekers. “trolls” and “reputed personalities” exist concurrently, thus, official agencies and media should consider the influence of these two types of users when controlling rumors.

## Rumor clarification and interaction model

### Model overview

Model 1 identifies the strategic interactions between three clusters of users in the context of rumor propagation and clarification. A decision-maker is defined as User A who has resources, authority, and responsibility to resist rumor, guard against falsehood spread of the truth, and maintain social network stability. Agencies such as official departments, social media companies, popular science platforms, and rumor refutation platforms are under the category of User A. In addition to considering the clarification strategies of official institutions, the behavior of the most influential users in social networks is also important. These influential people may help to clarify rumors. Conversely, they may also accelerate the spread of rumors. In this study, we classify influential users into trolls and reputed personalities according to different goals. Trolls(User B) are assumed to spread rumors to maximize their interest, regardless of whether those rumors are true or not. Reputed personalities(User C) are assumed to maximize their influence and social network credit ranking. They will decide how to participate based on their judgments on the authenticity of rumors and official behavior. The objective of this model is to study the impact of User A’s rumor clarification strategies on User B’s decision to propagate or terminate rumors and on User C’s choices of dissemination, support, opposition, and neutral participation. This is obtained by modeling the scenario of rumor clarification using a sequential game model.

### Notations, assumptions, and descriptions of the model

**FIGURE 1:**
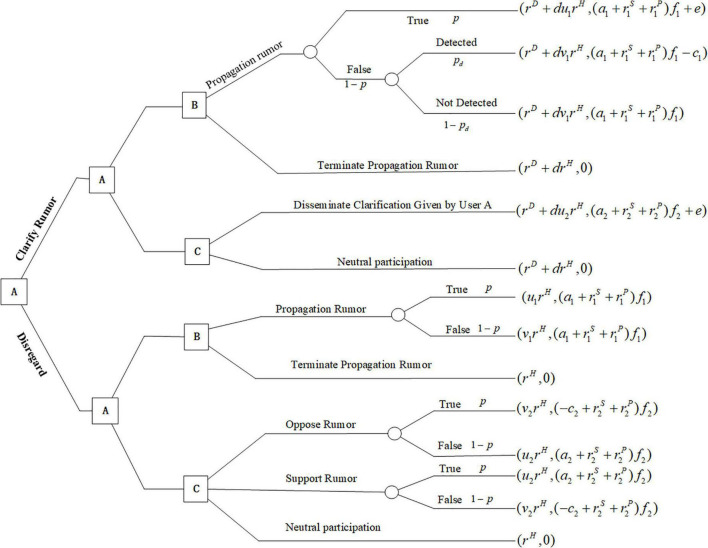
The sequence of moves of players in a rumor clarification and propagation game.

Notations used in this model are introduced and defined in Table 1, which includes three users’ decision options, three decision variables, nineteen parameters, and three functions. The sequence of player moves is shown in Figure 1. User A is assumed to be the leader in the game model, who can make a decision first on whether to clarify rumors. We assume that User A minimizes the expected loss *L*_1*A*_, User B maximizes the expected profit *M*_1*B*_, and User C maximizes the expected utility *U*_1*C*_. In this model, each player pursues different objectives for their distinct identities: User A seeks to minimize the cost of rumor control and the negative impact of rumor dissemination; User B seeks to maximize the profit by running advertisements or spreading misinformation on purpose; User C seeks to maximize his influence and credibility ratings in the social networks.

**TABLE 1:** Notations used in this model.

Decision options	
D, ND	User A: Clarify or Disregard rumor
P, T	User B: Propagate or Terminate propagating rumor
Q, K, S, N	User C: Disseminate clarification given by User A, Oppose or Support rumor, Neutral participation
**Decision variables**	
*x*_*i*_	Whether User A decides to choose option *i*,where *i* ∈ {*D*,*ND*} and *x*_*i*_ ∈ {0,1}
*y*_*j*_	Whether User B decides to choose option *j*,where *j* ∈ {*P*,*T*} and *y*_*j*_ ∈ {0,1}
*z*_*q*_	Whether User C decides to choose option *q*,where *q* ∈ {*Q*,*K*,*S*,*N*} and *z*_*q*_ ∈ {0,1}
**Parameters**	
*r^D^*	Cost of rumor clarification of User A
*r^H^*	Impact of rumor
*d*	Clarification index where *d* ∈ [0,1]
*p*	Probability that rumor is true where *p* ∈ [0,1]
*p*_*d*_	Probability that User B is detected to spread rumors where *p*_*d*_ ∈ [0,1]
*u*_1_,*u*_2_	Mitigation index of spreading correct information by User B/C where *u*_1_,*u*_2_ ∈ [0,1]
*v*_1_, *v*_2_	Deterioration index of propagating rumor by user B/spreading false information by User C where *v*_1_≥1, *v*_2_≥1
*f*_1_, *f*_2_	Number of followers of User B, User C
*a*_1_, *c*_1_	Profit/Cost (detected) from spreading rumors to each follower of User B
*e*	Reward from disseminating clarification when User A choose to clarify
*a*_2_, *c*_2_	Benefit/Cost from spreading correct/false information to each follower of User C
$r_{1}^{S}$, $r_{1}^{P}$	Participation rate obtained by User B due to importance/popularity of the event
$r_{2}^{S}$, $r_{2}^{P}$	Participation rate obtained by User C due to importance/popularity of the event
**Functions**	
*L*_1*A*_(*x*_*i*_,*y*_*j*_,*z*_*q*_)	Expected loss of User A
*M*_1*B*_(*x*_*i*_,*y*_*j*_)	Expected profit of User B
*U*_1*C*_(*x*_*i*_,*z*_*q*_)	Expected utility of User C

In this article, a rumor is considered to have a certain probability of being true, which is modeled by *p*. For the three users in the sequential game model, User A can choose to clarify (D) or disregard (ND) rumors. Given that User A chooses to clarify the rumor, User B can choose to propagate (P) or terminate propagating (T) the rumor, and User C can decide to disseminate (Q) rumor clarification information or neutrally participate (N). The strategy of rumor propagation by User B brings him profit *a*_1_*f*_1_; if the rumor is true, he can gain additional rewards *e* from the platform. If the rumor is false, he has a certain chance *p*_*d*_ to be detected by the platform and bears a cost *c*_1_ for spreading the rumor. No profit or cost is associated with the choice of terminating the propagation of rumors to User B. If User C disseminates to clarify information to their social network, he earns benefit *a*_2_*f*_2_ from spreading true information to his followers and platform rewards *e*, and no benefit or cost is produced if he does nothing. Given that User A chooses to disregard rumors, the strategy of User B remains unchanged. Because User C does not know whether the rumor is true or false, he may choose to support, oppose, or engage in neutral participation. User C earns benefit *a*_2_*f*_2_ if he spreads true information to his followers and bears the cost *c*_2_*f*_2_ if he spreads wrong news, no benefit or cost is produced if he neutrally participates.

For User A, there is a cost *r^D^* and impact *r^H^* if his choice is to clarify the rumor. If User A chooses to clarify the rumor, the influence of the rumor will be reduced to *dr^H^*. Additionally, if User B or C spreads the truth, and the impact of the rumor decreases by a factor *u*_1_ or *u*_2_. If User B or C spreads false information, the impact of the rumor increases by a factor *v*_1_*v*_2_. The participation rate of Users B and C in rumor propagation also depends on the importance and popularity of the event *r^S^*, *r^P^*.

In this model, the objective of User A is to minimize the expected cost *L*_1*A*_ by making strategies *x*_*i*_,*i* ∈ {*D*,*ND*} to clarify the rumor. The objective of User B is to maximize the expected profit *M*_1*B*_ by making a strategy *y*_*j*_,*j* ∈ {*P*,*T*} to spread rumors or not. The objective of User C is to maximize the expected utility *U*_1*C*_ by making a strategy *z*_*q*_, *q* ∈ {*Q*,*K*,*S*,*N*} to disseminate, support, oppose or engage in neutral participation. Thus, the optimal expression of the three players in the model can be described as follows:


(1) $$\begin{array}{l} \min L_{A}(x_{i},y_{j},z_{q})=x_{D}r^{D}+x_{D}(1-(1-u_{2})z_{Q})((pu_{1}\\ \;+(1-p)v_{1}-1)y_{P}+1))dr^{H}+(1-x_{D})\\ \;((pu_{1}+(1-p)v_{1}-1)y_{P}+1)((pv_{2}\\ \;+(1-p)u_{2}-1)z_{K}+(pu_{2}+(1-p)v_{2}-1)\\ \;z_{S}+1)r^{H} \end{array}$$

(2) $$\max M_{B}(x_{i},y_{j})=x_{D}y_{P}((a_{1}+r_{1}^{S}+r_{1}^{P})f+pe-(1-p)p_{d}c_{1})+(1-x_{D})y_{P}f_{1}(a_{1}+r_{1}^{S}+r_{1}^{P})$$

(3) $$\begin{array}{l} \max U_{C}(x_{i},z_{q})=x_{D}z_{Q}((a_{2}+r_{2}^{S}+r_{2}^{P})f_{2}+e)+(1-x_{D})z_{K}f_{2}\\ \;((1-p)a_{2}-pc_{2}+r_{2}^{S}+r_{2}^{P})+(1-x_{D})z_{S}f_{2}\\ \;(pa_{2}-(1-p)c_{2}+r_{2}^{S}+r_{2}^{P})) \end{array}$$

### Best responses of Users B and C

Since Users B and C are supposed to be the secondary movers in this model, the study first derives the best response functions for Users B and C in different situations, ${\hat{y}}_{n}$, ${\hat{z}}_{n}$. In addition, because User B’s decision is primarily based on the maximum profit rather than the authenticity of the rumor, only User C considers the authenticity of the rumor. Therefore, the probability of being detected to spread rumors *p*_*d*_ is used to distinguish the boundary conditions of different reaction strategies of User B, and the probability of the rumor being true *p* is used to distinguish the boundary conditions of different reaction strategies of User C, which are defined as follows:


(4) $${\hat{y}}_{n}\equiv \underset{y_{i\,j}\in \left\{0,1\right\}}{\arg\max}M_{1\,B}\,\left(x_{i},y_{i\,j}\right),\,w\,h\,e\,r\,e\,\,n=1,2$$

(5) $${\hat{z}}_{n}\equiv \underset{z_{i\,q}\in \left\{0,1\right\}}{\arg\max}U_{1\,C}\,\left(x_{i},z_{i\,q}\right),\,w\,h\,e\,r\,e\,\,n=1,2$$

**Proposition 1.** The best response of User B ${\hat{y}}_{n}$ is given by:

For *n* = 1,


(6) $${\hat{y}}_{n}\equiv \left\{ \begin{array}{c} P\;i\,f\,p_{d}\leq \frac{\left(a_{1}+r_{1}^{S}+r_{1}^{P}\right)\,f_{1}+p\,e}{\left(1-p\right)\,c_{1}}\\ T\;i\,f\,p_{d}\geq \frac{\left(a_{1}+r_{1}^{S}+r_{1}^{P}\right)\,f_{1}+p\,e}{\left(1-p\right)\,c_{1}} \end{array}\right.$$

For *n* = 2, strategy P always takes precedence over T, therefore, ${\hat{y}}_{n}\equiv P$.

**Proposition 2.** The best response of User C, ${\hat{z}}_{n}$, is given by:

For *n* = 1, strategy Q always takes precedence over N, therefore, ${\hat{z}}_{n}\equiv Q$.

For *n* = 2,


(7) $${\hat{z}}_{n}\equiv \left\{ \begin{array}{c} S\;i\,f\,p\geq \max\left(\frac{1}{2},\frac{c_{2}-\left(r_{2}^{S}+r_{2}^{P}\right)}{a_{2}+c_{2}}\right)\\ K\;i\,f\,p\leq \min\left(\frac{1}{2},\frac{a_{2}+\left(r_{2}^{S}+r_{2}^{P}\right)}{a_{2}+c_{2}}\right)\,\\ N\;i\,f\,p\in \left(\frac{a_{2}+\left(r_{2}^{S}+r_{2}^{P}\right)}{a_{2}+c_{2}},\frac{c_{2}-\left(r_{2}^{S}+r_{2}^{P}\right)}{a_{2}+c_{2}}\right) \end{array}\right.$$

**Remark. Proposition 1** and **Proposition 2** define the boundary conditions of different reaction strategies of Users B and C. To explore the influence of various parameters on the best response of Users B and C, numerical simulation is used to describe different conditions. The baseline parameters in this model are: *p* = 0.5, *p*_*d*_ = 0.9, *a*_1_ = 1, *a*_2_ = 0.8, *c*_1_ = 9, *c*_2_ = 2, *e* = 1.5, $r_{1}^{S}=0.4$, $r_{1}^{P}=0.3$, $r_{2}^{S}=0.3$, $r_{2}^{P}=0.2$, *f*_1_ = 2, *f*_2_ = 2, *r^D^* = 7.0, *r^H^* = 5.0, *d* = 0.75, *u*_1_ = 0.5, *u*_2_ = 0.6, *v*_1_ = 1.5, and *v*_2_ = 1.3. In reality, these parameters can be determined through the mechanism of online social media and the rumor spread data. Specifically, the probability *p* of a rumor being true can be obtained from the historical database of the rumors according to its framework, content, emotion, and rationality. The probability *p*_*d*_ that User B is detected to continue spreading rumors after User A releases clarification information is determined by the supervision mechanism of the social platform. The revenue *a*_*i*_ and rumor mitigation index *u*_*i*_ of Users B and C can be calculated from the average number of likes, shares, positive comments, and advertising fees obtained. The loss value *c*_2_ and rumor deterioration index *v*_*i*_ of Users B and C can be determined from the average number of negative comments due to the dissemination of error information. In particular, to prevent trolls from spreading rumors and to encourage reputed personalities to spread correct information, a large penalty value *c*_1_ and additional rewards *e* are set. In addition, $r_{i}^{S}$ and $r_{i}^{P}$ can be obtained based on the average number of likes, shares, and positive comments obtained by users B and C due to the importance of events and the popularity of rumor disseminators. The a number of followers *f*_*i*_ can be obtained from the user profile of the microblogging platform. The primary focus of User A is the cost of rumors clarification and the impact of rumors on social networks. The cost of clarifying rumors *r^D^* is primarily determined by the rumor refuting means, the type of resources used by User A, and the conditions of specific rumor cases. The impact of rumors *r^H^* can be quantified through relevant public opinion surveys. The rumor clarification index *d* can be obtained by comparing the amount of true or false information shared before and after the official rumor clarification.

**FIGURE 2:**
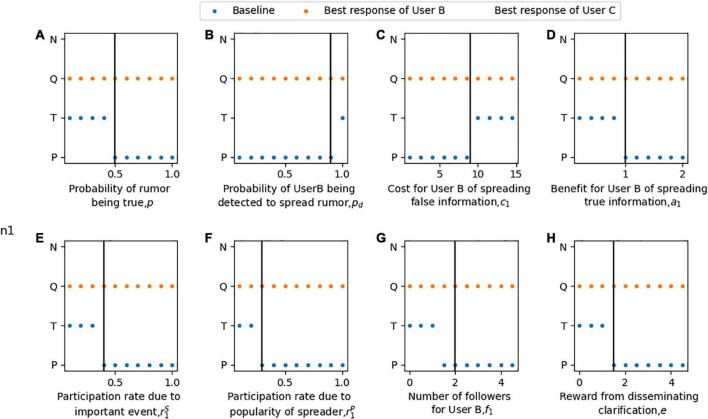
The best response of User B and User C gave that User A clarified the rumor in the rumor clarification model. For part panel **(A–H)**, the x-axis represents the change of each parameter, the y-axis indicates the optimal choice of Users B and C with the change of parameters when given that User A chooses to clarify rumors. Blue indicates the strategy P or T of User B. Orange indicates the strategy Q or N of user C. The vertical bar indicates the baseline value of each parameter.

**FIGURE 3:**
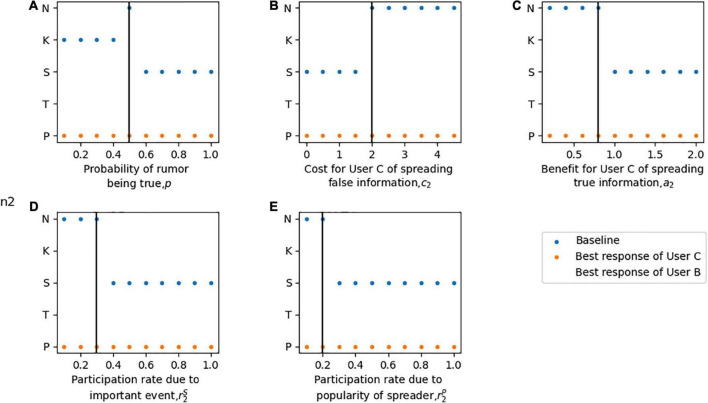
The best response of Users B and C gave that User A disregards the rumor in the rumor clarification model. For part panel **(A–E)**, the x-axis represents the change of each parameter, the y-axis indicates the optimal choice of Users B and C with the change of parameters when given that User A chooses not to clarify rumors. Orange indicates the strategy P or T of User B. Blue indicates the strategy S, K or N of User C. The vertical bar indicates the baseline value of each parameter.

The boundary conditions for these response strategies of Users B and C are shown in Figures 2, 3. As shown in Figures 2A–H, when User A chooses the strategy of clarifying the rumor (*n* = 1), User B may propagate or terminate propagation rumors. Under this condition, User C’s optimal strategy is always to disseminate clarification information. Figure 2A shows that when the probability of the rumor being true is high, User B will continue to spread the case after User A publishes the clarification information. In contrast, when the probability of the rumor being true is low, User B will choose to terminate the dissemination after the rumor is clarified. As shown in Figure 2B, the detection accuracy of malicious spreading rumors must be sufficiently high before User B chooses to stop spreading them. In Figures 2C,D, User B seeks advantages and avoids disadvantages: he thus chooses to spread rumors when the benefit of spreading rumors is high, while the huge cost stops him from spreading wrong information. Figures 2E,F show that a higher engagement rate due to the importance of the event or popularity of the spreader motivates User B to change his strategy from termination to propagation. Figure 2G shows that the more followers User B has, the more active he is in spreading rumors. In Figure 2H, when there are more rewards for spreading clarification information, the best choice for User B will also change from terminating to spreading rumors.

Conversely, as shown in Figures 3A–E, if User A chooses to disregard the rumor (*n* = 2), User B will always choose to spread rumors without considering the authenticity of rumors. User C may support, oppose or participate in the rumor neutrally because the fact is unknown. As shown in Figure 3A, when the probability that the rumor is true is low, User C chooses to oppose the rumor. On the contrary, when the rumor is more likely to be the truth, he shifts his strategy to support the rumor. Figures 3B,C show that when the benefit of spreading true information or the cost of propagating false information is low, User C engages in neutral participation, while a higher benefit or lower cost motivates him to support rumor spreading. In Figures 3D,E, the high participation rate urges User C to change his strategy from neutral participation to supporting rumors due to the importance of the event or the popularity of the disseminator. User C’s strategies toward rumors should be supportive or opposing according to the rumor authenticity probability. However, User C only selects neutral participation or support for rumors in Figures 3A–E, which have been affected by the baseline value.

### Nash equilibrium solutions

Definition 1. A set of User A’s, User B’s, and User C’s optimal strategies (x*,y*,z*) is called a Subgame-Perfect Nash Equilibrium (SPNE)(Agarwal et al., 2022) if and only if:


(8) $$x^{*}=\underset{x\in \left\{0,1\right\}}{\arg\max L_{A}\,\left(x,{\hat{y}}_{n},{\hat{z}}_{n}\right)},w\,h\,e\,r\,e\,n\in \left\{1,2\right\}$$

(9) $$y^{*}={\hat{y}}_{n}\,\left(x^{*}\right)\,\underset{y_{n}\in \left\{0,1\right\}}{=\arg\max M_{B}\,\left(x^{*},y_{n}\right)},w\,h\,e\,r\,e\,n\in \left\{1,2\right\}$$

(10) $$z^{*}={\hat{z}}_{n}\,\left(x^{*}\right)\,\underset{z_{n}\in \left\{0,1\right\}}{=\arg\max U_{C}\,\left(x^{*},z_{n}\right)},w\,h\,e\,r\,e\,n\in \left\{1,2\right\}$$

**Proposition 3.** The SPNE values of the clarification strategies model of rumors along with the optimal expected loss, profit, and utility of every player are exhibited in Table 2, where *R*_*m*_, *m* = 1,2,…,5 are the best cases defined in Supplementary Appendix A.3$L_{1\,A}^{*}$, $M_{1\,B}^{*}$ and $U_{1\,C}^{*}$ are the optimal expected loss, profit, and utility for User A, User B, and User C, respectively.

**TABLE 2:** Equilibrium values of the rumor clarification model.

Cases	(x*,y*,z*)	$L_{1\,A}^{\text{*}}$	$M_{1\,B}^{\text{*}}$	$U_{1\,C}^{\text{*}}$
*R*_1_	(*D*,*P*,*Q*)	*r^D^* + (*pu*_1_ + (1−*p*)*v*_1_)*u*_2_*dr^H^*	$\left(a_{1}+r_{1}^{S}+r_{1}^{P}\right)\,f_{1}+p\,e-\left(1-p\right)\,p_{d}\,c_{1}$	$\left(a_{2}+r_{2}^{S}+r_{2}^{P}\right)\,f_{2}+e$
*R*_2_	(*D*,*T*,*Q*)	*r^D^* + *u*_2_*dr^H^*	0	$\left(a_{2}+r_{2}^{S}+r_{2}^{P}\right)\,f_{2}+e$
*R*_3_	(*ND*,*P*,*S*)	(*pu*_1_ + (1−*p*)*v*_1_)⋅(*pu*_2_ + (1−*p*)*v*_2_)*r^H^*	$\left(a_{1}+r_{1}^{S}+r_{1}^{P}\right)\,f_{1}$	$\left(p\,a_{2}-\left(1-p\right)\,c_{2}+r_{2}^{S}+r_{2}^{P}\right)\,f_{2}$
*R*_4_	(*ND*,*P*,*K*)	(*pu*_1_ + (1−*p*)*v*_1_)⋅(*pv*_2_ + (1−*p*)*u*_2_)*r^H^*	$\left(a_{1}+r_{1}^{S}+r_{1}^{P}\right)\,f_{1}$	$\left(\left(1-p\right)\,a_{2}-p\,c_{2}+r_{2}^{S}+r_{2}^{P}\right)\,f_{2}$
*R*_5_	(*ND*,*P*,*N*)	*r^H^*	$\left(a_{1}+r_{1}^{S}+r_{1}^{P}\right)\,f_{1}$	0

*Indicates the optimal strategies under subgame-perfect Nash equilibrium.

**Remark. Proposition 3** represents five possible SPNE strategies for three players. User A chooses to clarify rumors (x* = *D*) at equilibrium in cases 1 and 2 and disregards rumors (x* = *ND*) in cases 3, 4, and 5. User B chooses to terminate propagating rumors (y* = *T*) at equilibrium in case 2, and spreading rumors(y* = *P*) in other cases. User C disseminates the clarification information (z* = *Q*) at equilibrium in cases 1 and 2, supports the rumors (z* = *S*) in case 4, opposes (z* = *K*) in case 3, and engages in neutral participation (z* = *N*) in case 5.

### Sensitive analyzes of equilibrium solutions

**FIGURE 4:**
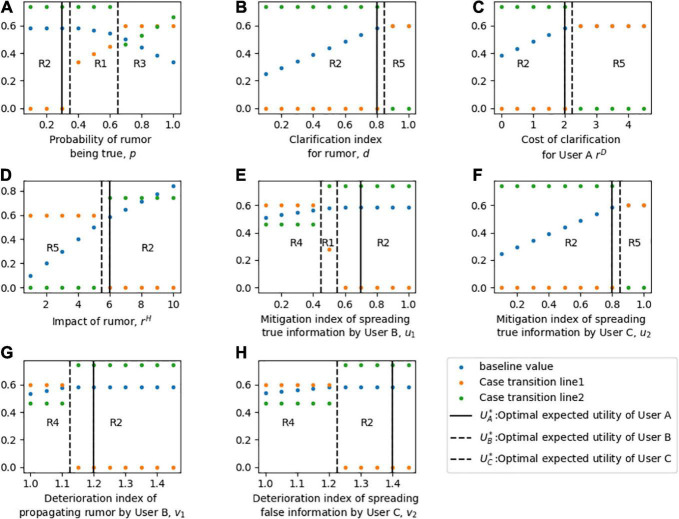
Sensitivity analysis of the optimal strategies and expected utilities of three players. For part panel **(A–H)**, the x-axis represents the change of each parameter, the y-axis indicates the sensitivity of the equilibrium. R1-R5 in the part labels represents the different cases of strategy combination. The solid vertical line represents the baseline value of each parameter, and the dotted vertical line represents the transition of each Case.

In this section, the sensitivity of each parameter to the equilibrium solution of three players is discussed. To compare the objective functions of the three players, the expected loss function of User A *L*_1*A*_ and the expected profit function of User B *M*_1*B*_ is converted into expected utility functions *U*_1*A*_ and *U*_1*B*_ respectively. In the sensitivity analysis, the optimal expected utility of User A is the first echelon to be considered because he is the leader who gives priority to decision-making, and the expected utility of the other two players is considered in the second step. The sensitivity analysis of parameters to the equilibrium solution is shown in Figure 4.

Figure 4A shows the sensitivity of the equilibrium solution to the probability *p* of the rumor being true. When *p* is low (i.e., the rumor is easily recognized as false), User A chooses to publish the rumor clarification information, User B chooses not to spread the rumor for fear of the risk of high loss of interest caused by being detected to propagating rumor, and User C chooses to follow User A to disseminate the clarification information to his social network. When *p* is moderate, and the authenticity of the rumor is difficult to judge, the equilibrium strategy of User A is to clarify the rumor; User B will take advantage of the fuzziness of the information and spread the rumor to make a profit; and the equilibrium decision of User C remains unchanged. With a higher *p*, User A chooses to disregard the rumor, and User B chooses to propagate it to make a profit. At this time, without the participation of official agencies, User C chooses to support the rumor due to the high authenticity of the news.

Figures 4B,C show the sensitivity of the equilibrium behaviors to parameters *dr^D^*. At low *d* or *r^D^*, the optimal strategy of User A is to clarify the rumor. Because lower *d* or *r^D^* means that User A clarifies rumors at a lower cost and higher quality. In addition, with low *d* or *r^D^*, User B chooses not to spread rumors, and User C chooses to disseminate clarification information given by User A. In contrast, with high *d*, *r^D^*, which means that the cost of rumor clarification is too high or the clarification effect is poor, User A changes his equilibrium behavior to disregard the rumor. Higher *d* and *r^D^* motivate User B to spread rumors and motivate User C to Participate neutrally.

In Figure 4D, it is observed that a low *r^H^* motivates User A to disregard the rumor, while a high *r^H^* motivates him to clarify it. For User B, when *r^H^* is low, he chooses to spread rumors for profit. However, when the influence of rumors *r^H^* is too high, he will change his strategy to stop spreading rumors, because User A will also impose high penalties for the malicious spreading of rumors when clarifying rumors. Similarly, when *r^H^* increases, the equilibrium strategy of User C changes from neutral participation to disseminating clarification published by User A.

Figures 4E–G illustrate the sensitivity of equilibrium strategies in regard to values of mitigation and deterioration indices *u*_1_*v*_1_, respectively. At low *u*_1_ and *v*_1_, User A chooses to disregard rumors, User B chooses to spread rumors, and the strategy of User C is to oppose the rumors given that the baseline is *p* = 0.3. At higher *u*_1_ and *v*_1_, User A translates his decisions to clarify it and User C chooses to follow User A. Unlike Users A and B, with changes *u*_1_, User B will choose to spread the rumor first, then terminate it, and then continue to spread it.

Figures 4F,H describe how sensitive the equilibrium decisions are when considering parameters *u*_2_*v*_2_. A low *u*_2_ and a high *v*_2_ motivate User A to clarify rumors, motivate User B to terminate spreading rumors, and motivate User C to publish clarification information given by User A. While a high *u*_2_ and a low *v*_2_ motivate User A to disregard the rumor, motivate User B to spread the rumor. Significantly, a high *u*_2_ motivates User C to participate neutrally, which may be due to the limited role that User C believes he can play. While a low *v*_2_ motivates User C to oppose the rumor, given that the baseline v *p* is low.

### Research findings

The attention caused by false information is much higher than that of real information. Therefore, when the probability of rumors being true is low, User A chooses to clarify the event, and User B chooses not to spread rumors due to the high risk of being punished for spreading false information, which also motivates User C to disseminate the clarification information given by User A. When the influence of a rumor is high, the cost of publishing false information is also high, and the official’s control is stricter. For User B, the profits of spreading rumors are attractive, which in return is accompanied by the high risk of being severely punished. For User C, if there is no official rumor refutation, he will choose to support, oppose or remain neutral when participating in the spread of the rumor according to his understanding of the rumor. If he chooses to support false rumors, it will aggravate the impact of rumors on social networks and increase the cost associated with the control of rumor propagation of User A. Users B and C are also motivated to participate based on the importance and popularity of rumors. This model serves as a decision-making tool for User A to make critical strategies on whether to clarify rumors by considering the cost and impact of rumors. In addition, the strategy for User A to clarify rumors also depends on whether User B chooses to spread them and whether User C spreads correct or wrong information to his social network.

In fact, User B tends to spread rumors based on “loophole advantage” to make a profit before the public knows the truth, while User C spreads information to his followers to provide opinions. To prevent User B from maliciously spreading rumors and User C from spreading false information due to unknown truth, User A should verify the truth of the rumor before publishing clarification information that can reduce the uncertainty of the rumor, so that User B has no exploitable vulnerabilities and User C can make wise decisions. With this motivation, Model 2 is developed to reduce the impact of uncertain information onuser judgment and rumors on social networks.

## Rumor verification and interaction model

### Model overview

Once a rumor case is detected in a social media network, relevant actions to block its dissemination must be taken by departments involved to reduce social losses. However, rapid clarification of rumors may not work in some cases, and unverified information may leave room for speculation and lead to serious harmful effects. For example, people with ulterior motives may use the loophole and spread rumors to profit, and some positive influencers may release false information due to ambiguity, thus accelerating the spread of rumors. Therefore, it is particularly important to determine the balance between rapid response and the amount of time, effort, and money to verify rumor information before clarification. Model 2 defines the equilibrium strategy for User A so that he can minimize the impact of rumors through the trade-off between rapid response with partial information or postprocessing after verifying information, and then discusses the strategic interactions between Users A, B, and C before and after rumor verification. The objective of Model 2 is to determine the best strategy for User A to verify the information and to solve the problem where User B uses the “loophole advantages” and User C makes incorrect choices due to unclear rumor information. This situation is obtained by modeling the scenario of rumor verification and clarification by a sequential game model.

### Notations, assumptions, and descriptions of the model

**FIGURE 5:**
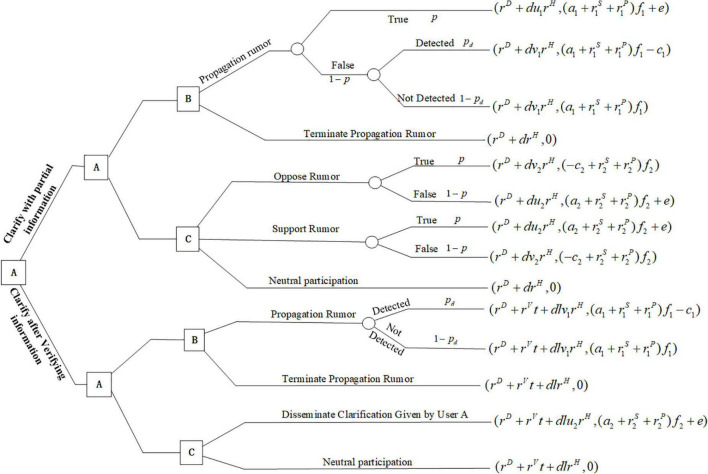
The sequence of players moves in a rumor verification and clarification game.

Because the purpose of Model 2 is to consider the impact of verification before rumor clarification, and the notation assumption is basically consistent with Model 1, only notations different from Model 1 are introduced and defined in Table 3. The sequence of moves of the three players is shown in Figure 5.

**TABLE 3:** Notations different from Model 1 in Model 2.

Decision options	
D	User A:Clarify with partial information
VD	User A:Clarify after verifying information
**Decision variables**	
*x*_*i*_	Whether User A decides to choose option *i*,where *i* ∈ {*D*,*VD*} and *x*_*i*_ ∈ {0,1}
**Parameters**	
*r^V^*	Verification cost per unit time of User A
*l*	Verification index where *l* ∈ [0,1]
*t*	Verifying period of User A
**Functions**	
*L*_2*A*_(*x*_*i*_,*y*_*j*_,*z*_*q*_)	Expected loss of User A
*M*_2*B*_(*x*_*i*_,*y*_*j*_)	Expected profit of User B
*U*_2*C*_(*x*_*i*_,*z*_*q*_)	Expected utility of User C

In this section, User A is assumed to be the leader in the sequential game model, who can make a decision first on clarification with partial information(D) or clarify the fact after verifying information (VD). If User A chooses to clarify rumors quickly with partial unverified information, User B chooses to propagate(P) or terminate propagating(T) rumors the same as Model 1. However, contrary to Model 1, User C in Model 2 will make his own decision and not completely believe the judgment of User A (i.e., oppose (K), support (S), or engage in neutral participation (N)). In this case, the expected loss, profit, and utility of the three players are similar to those in Model 1. Given that User A chooses to obtain verified information for rumor clarification.

For User A, a time-varying cost *r^V^t* exists during the verification period, where *r^V^* is the verification cost per unit time and *t* is the verification period. Publishing verified information by User A will mitigate the impact of rumors *dlr^H^*, where *d* is the mitigation index of clarifying the rumor directly and *l* is the verification index of the rumor. In this situation, User B may still spread false information at the risk of severe punishment, but it is more likely that he chooses to stop spreading rumors. User C can decide to disseminate (Q) this rumor clarification information or may engage in neutral participation (N).

In this model, the objective of User A is to minimize the expected cost *L*_2*A*_ by making strategies *x*_*i*_,*i* ∈ {*D*,*VD*} to clarify partial information or verify it before clarification. The objective of User B is to maximize the expected profit *M*_2*B*_ by making a strategy *y*_*j*_,*j* ∈ {*P*,*T*} to spread rumors or not. The objective of User C is to maximize the expected utility *U*_2*C*_ by making a strategy *z*_*q*_, *q* ∈ {*Q*,*K*,*S*,*N*} to disseminate, support, oppose or engage in neutral participation. Thus, the optimal expression of the three players in the model can be shown as follows:


(11) $$\begin{array}{l} \min L_{2A}(x_{i},y_{j},z_{q})=r^{D}+x_{D}((pu_{1}+(1-p)v_{1}-1)y_{P}+1)\\ \;((pv_{2}+(1-p)u_{2}-1)z_{K}+(pu_{2}+(1-p)\\ \;v_{2}-1)z_{S}+1)dr^{H}+(1-x_{D})(r^{V}t+(1-\\ \;(1-u_{2})z_{Q})(1+(v_{1}-1)y_{P})dlr^{H}) \end{array}$$

(12) $$\max M_{2B}(x_{i},y_{j})=x_{D}y_{P}((a_{1}+r_{1}^{S}+r_{1}^{P})f_{1}+pe-(1-p)p_{d}c_{1})+(1-x_{D})y_{P}((a_{1}+r_{1}^{S}+r_{1}^{P})f_{1}-p_{d}c_{1})$$

(13) $$\begin{array}{l} \max U_{2C}(x_{i},z_{q})=x_{D}(z_{K}(((1-p)a_{2}-pc_{2}+r_{2}^{S}+r_{2}^{P})f_{2}+\\ \;(1-p)e)+z_{S}((pa_{2}-(1-p)c_{2}+r_{2}^{S}+r_{2}^{P})\\ \;f_{2}+pe))+(1-x_{D})z_{Q}((a_{2}+r_{2}^{S}+r_{2}^{P})\\ \;f_{2}+e) \end{array}$$

### Best responses of Users B and C

In Model 2, it is assumed that User B and User C are the followers, thus, their best response functions (${\hat{y}}_{n}$, ${\hat{z}}_{n}$) in different situations are derived first, which are defined as below:


(14) $${\hat{y}}_{n}\equiv \underset{y_{i\,j}\in \left\{0,1\right\}}{\arg\max}M_{2\,B}\,\left(x_{i},y_{i\,j}\right),\,w\,h\,e\,r\,e\,\,n=1,2$$

(15) $${\hat{z}}_{n}\equiv \underset{z_{i\,q}\in \left\{0,1\right\}}{\arg\max}U_{2\,C}\,\left(x_{i},z_{i\,q}\right),\,w\,h\,e\,r\,e\,\,n=1,2$$

**Proposition 4.** The best response of User B, ${\hat{y}}_{n}$, is given by:

For *n* = 1,


(16) $${\hat{y}}_{n}\equiv \left\{ \begin{array}{c} P\;i\,f\,p_{d}\leq \frac{\left(a_{1}+r_{1}^{S}+r_{1}^{P}\right)\,f_{1}+p\,e}{\left(1-p\right)\,c_{1}}\\ T\;i\,f\,p_{d}\geq \frac{\left(a_{1}+r_{1}^{S}+r_{1}^{P}\right)\,f_{1}+p\,e}{\left(1-p\right)\,c_{1}} \end{array}\right.$$

For *n* = 2,


(17) $${\hat{y}}_{n}\equiv \left\{ \begin{array}{c} P\;i\,f\,p_{d}\leq \frac{\left(a_{1}+r_{1}^{S}+r_{1}^{P}\right)\,f_{1}}{c_{1}}\\ T\;i\,f\,p_{d}\geq \frac{\left(a_{1}+r_{1}^{S}+r_{1}^{P}\right)\,f_{1}}{c_{1}} \end{array}\right.$$

**Proposition 5.** The best response of User C, ${\hat{z}}_{n}$, is given by:

For *n* = 1,


(18) $${\hat{z}}_{n}\equiv \left\{ \begin{array}{c} S\;i\,f\,p\geq \max\left(\frac{1}{2},\frac{\left(c_{2}-r_{2}^{S}-r_{2}^{P}\right)\,f_{2}}{\left(a_{2}+c_{2}\right)\,f_{2}+e}\right)\\ K\;i\,f\,p\leq \min\left(\frac{1}{2},\frac{\left(a_{2}+r_{2}^{S}+r_{2}^{P}\right)\,f_{2}+e}{\left(a_{2}+c_{2}\right)\,f_{2}+e}\right)\,\\ N\;i\,f\,p\in \left(\frac{\left(a_{2}+r_{2}^{S}+r_{2}^{P}\right)\,f_{2}+e}{\left(a_{2}+c_{2}\right)\,f_{2}+e},\frac{\left(c_{2}-r_{2}^{S}-r_{2}^{P}\right)\,f_{2}}{\left(a_{2}+c_{2}\right)\,f_{2}+e}\right) \end{array}\right.$$

For *n* = 2, strategy Q always takes precedence over N, therefore, ${\hat{z}}_{n}\equiv Q$.

**Remark. Proposition 4** and **Proposition 5** define the boundary conditions of different reaction strategies of Users B and C. When User A chooses to quickly clarify the rumor according to partial information currently available (*n* = 1), User B may choose to spread (P) or terminate spreading (T) the rumor. User C may choose among three optimal strategies: support (S), oppose (K), or engage in neutral participation(N). Conversely, when User A chooses to clarify the fact after verifying information (*n* = 2), User B may still choose to spread (P) or stop spreading (T) the rumor, but the boundary conditions are different from those in the case of *n* = 1. User C’s optimal strategy is to disseminate the clarification information to his social network (Q).

### Nash equilibrium solutions

**Proposition 6.** The SPNE values of the rumor verification and clarification strategies model, as well as the optimal expected loss, profit, and utility of players are exhibited in Table 4, where *V*_*m*_, *m* = 1,2,…,8 are the optimal cases defined in Supplementary Appendix A.6. $L_{2\,A}^{*}$, $M_{2\,B}^{*}$ and $U_{2\,C}^{*}$ are the optimal expected loss, profit, and utility for Users A, B, and C, respectively.

**TABLE 4:** Equilibrium values of the rumor verification model.

Cases	(x*,y*,z*)	$L_{2\,A}^{\text{*}}$	$M_{2\,B}^{\text{*}}$	$U_{2\,C}^{\text{*}}$
*V*_1_	(*D*,*P*,*S*)	*r^D^* + (*pu*_1_ + (1−*p*)*v*_1_)(*pu*_2_ + (1−*p*)*v*_2_)*dr^D^*	$\left(a_{1}+r_{1}^{S}+r_{1}^{P}\right)\,f_{1}+p\,e-\left(1-p\right)\,p_{d}\,c_{1}$	$\left(p\,a_{2}-\left(1-p\right)\,c_{2}+r_{2}^{S}+r_{2}^{P}\right)\,f_{2}+p\,e$
*V*_2_	(*D*,*P*,*K*)	*r^D^* + (*pu*_1_ + (1−*p*)*v*_1_)⋅(*pv*_2_ + (1−*p*)*u*_2_)*dr^D^*	$\left(a_{1}+r_{1}^{S}+r_{1}^{P}\right)\,f_{1}+p\,e-\left(1-p\right)\,p_{d}\,c_{1}$	$\left(\left(1-p\right)\,a_{2}-p\,c_{2}+r_{2}^{S}+r_{2}^{P}\right)\,f_{2}+\left(1-p\right)\,e$
*V*_3_	(*D*,*P*,*N*)	*r^D^* + (*pu*_1_ + (1−*p*)*v*_1_)*dr^D^*	$\left(a_{1}+r_{1}^{S}+r_{1}^{P}\right)\,f_{1}+p\,e-\left(1-p\right)\,p_{d}\,c_{1}$	0
*V*_4_	(*D*,*T*,*S*)	*r^D^* + (*pu*_2_ + (1−*p*)*v*_2_)*dr^D^*	0	$\left(p\,a_{2}-\left(1-p\right)\,c_{2}+r_{2}^{S}+r_{2}^{P}\right)\,f_{2}+p\,e$
*V*_5_	(*D*,*T*,*K*)	*r^D^* + (*pv*_2_ + (1−*p*)*u*_2_)*dr^D^*	0	$\left(\left(1-p\right)\,a_{2}-p\,c_{2}+r_{2}^{S}+r_{2}^{P}\right)\,f_{2}+\left(1-p\right)\,e$
*V*_6_	(*D*,*T*,*N*)	*r^D^* + *dr^D^*	0	0
*V*_7_	(*VD*,*P*,*Q*)	*r^D^* + *r^V^t* + *v*_1_*u*_2_*dlr^D^*	$\left(a_{1}+r_{1}^{S}+r_{1}^{P}\right)\,f_{1}-p_{d}\,c_{1}$	$\left(a_{2}+r_{2}^{S}+r_{2}^{P}\right)\,f_{2}+e$
*V*_8_	(*VD*,*T*,*Q*)	*r^D^* + *r^V^t* + *u*_2_*dlr^D^*	0	$\left(a_{2}+r_{2}^{S}+r_{2}^{P}\right)\,f_{2}+e$

*Indicates the optimal strategies under subgame-perfect Nash equilibrium.

**Remark. Proposition 6** represents eight possible SPNE strategies for three players. User A chooses to clarify rumors quickly with partial information currently owned (x* = *D*) at equilibrium in cases 1-6, and clarifies it after verifying information (x* = *VD*) in cases 7 and 8. User B chooses to spread rumors (y* = *P*)in cases 1, 2, 3, and 7, to terminate propagating rumors (y* = *T*) at equilibrium in cases 4, 5, 6, and 8. User C disseminates the clarification information (z* = *Q*) at equilibrium in cases 7 and 8; supports the rumors (z* = *S*) in cases 1 and 4; opposes (z* = *K*) in cases 2 and 5; engages in neutral participation (z* = *N*) in cases 3 and 6.

### Sensitive analyzes of equilibrium solutions

**FIGURE 6:**
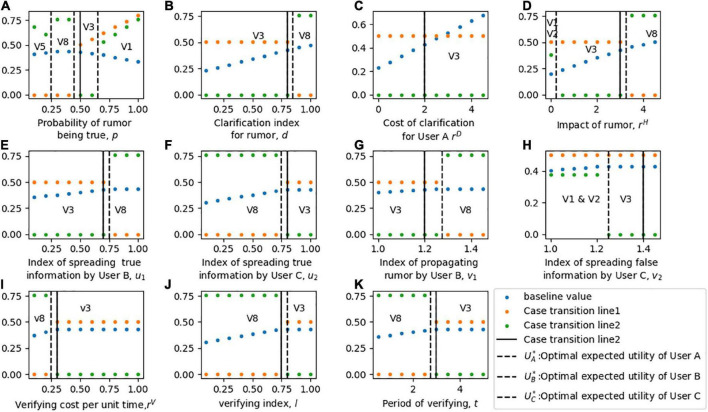
Sensitivity analysis of the optimal strategies and expected utilities of three players in Model 2. For part panel **(A–K)**, the x-axis represents the change of each parameter, the y-axis indicates the sensitivity of the equilibrium solution. V1-V8 in the part labels represents the different cases of strategy combination. The solid vertical line represents the baseline value of each parameter, and the dotted vertical line represents the transition of each Case.

In this section, the sensitivity of each parameter to the equilibrium solution of three players is discussed. To compare the objective functions of the three players, the expected loss function of User A *L*_2*A*_ and the expected profit function of User B *M*_2*B*_ is converted into expected utility functions *U*_2*A*_ and *U*_2*B*_ respectively. In the process of sensitivity analysis, the optimal expected utility of User A is the first echelon to be considered, and the expected utility of the other two players is considered in the second step. Numerical simulation is used to describe different conditions and the baseline values of parameters in this model can be described as: *p* = 0.5, *p*_*d*_ = 0.99, *a*_1_ = 0.3, *a*_2_ = 0.4, *c*_1_ = 5, *c*_2_ = 0.7, *e* = 1, $r_{1}^{S}=0.7$, $r_{1}^{P}=0.4$$r_{2}^{S}=0.4$, $r_{2}^{P}=0.3$, *f*_1_ = 5, *f*_2_ = 6, *u*_1_ = 0.7, *u*_2_ = 0.8, *v*_1_ = 1.2, *v*_2_ = 1.4*r^D^* = 2, *r^H^* = 3, *r^V^* = 0.3, *d* = 0.8, *l* = 0.75, and *t* = 3. A sensitivity analysis of parameters to the equilibrium solution is shown in Figure 6.

Figure 6A shows the sensitivity of the equilibrium behaviors of three players with respect to the parameter ***p***. User A chooses to clarify the rumor quickly with partial information at a low value of high ***p*** (i.e., the strategy of disregarding rumors is not considered in Model 2). A moderate ***p*** will encourage User A to obtain verified information. Given the setting of the baseline value, User B chooses not to spread the rumor when ***p*** is low and changes his strategy to spread the rumor at a high ***p***. For User C, when User A chooses to quickly clarify the rumor, he makes a decision of opposition, support, or neutral participation based on the probability of the rumor being true. When User A chooses to verify the rumor information, User C’s optimal behavior is to disseminate the clarification information to his social network.

In Figure 6B, the sensitivity of equilibrium decisions is illustrated relative to the parameter *d*. With a low *d*, User A chooses to clarify the rumor immediately, while User B chooses to spread the rumor regardless of the risk of punishment, and User C chooses neutral participation, given that the baseline *p* is moderate. At a high *d*, which means that the quality of clarifying quickly is poor, User A chooses to verify the information before sending clarification to the social network. In this case, User B terminates spreading rumors, and User C publishes the clarification information given by User A.

Figures 6C,D describe the equilibrium strategy of each player with respect to parameters *r^D^* and *r^H^*. Figure 6C shows that the cost of User A clarifying rumors does not affect the equilibrium solution with the given baseline setting; thus, the optimal solution of the players is (*D*,*P*,*N*). As shown in Figure 6D, a low *r^H^* motivates User A to clarify the rumor quickly with partial information currently available, while a higher *r^H^* motivates him to verify the detailed information of the rumor. For User B, when User A chooses to quickly clarify the rumor, he makes a decision to spread the rumor. When User A chooses to verify the rumor information, his optimal behavior is to terminate spreading the rumor. For User C, at an extremely low *r^H^*, the optimal benefits of supporting and opposing rumors are the same; at a moderate *r^H^*, he chooses neutral participation; and at a high *r^H^*, he chooses to follow User A and disseminate the clarification.

Figures 6E–H show the sensitivity of equilibrium behaviors in regard to the mitigation and deterioration index of User B and User C. In Figures 6E,G, at low *u*_1_ and *v*_1_, User A chooses to clarify rumors quickly, User B chooses to spread rumors, and the strategy of User C is to engage in neutral participation given the baseline *p* = 0.5. At higher *u*_1_ and *v*_1_, which means that the impact of the rumor is increased, User A translates his strategy to verify it before publishing clarification, User C chooses to terminate spreading and User C chooses to follow User A. Figures 6F,H describe how sensitive the equilibrium decisions are when considering parameters *u*_2_ and *v*_2_. A low *u*_2_ shows that User C has a strong impact on rumors, thereby acting as a factor of motivation for User A to verify the information. A high *u*_2_ and *v*_2_ increases the impact on social networks of rumor events; thus, User A chooses to clarify it quickly to avoid panic. User C chooses neutral participation considering that the positive impact is tiny and the negative impact is enormous.

Figures 6I–K analyze the sensitivity of equilibrium solutions with respect to variations in *r^V^*, *l* and *t*. These three parameters have similar effects on players’ decision-making. At low *r^V^*, *l* and *t*, User A is likely to spend time and energy in verifying the rumored case to gain verified information before clarifying it. Conversely, User A is motivated to switch his strategy to clarify quickly with partial information at high *r^V^*, *l* and *t*.

### Research findings

When the authenticity of a rumor is vague or its impact is high, the numerical analysis shows that the equilibrium strategy of User A is to verify the information and to obtain detailed verified information about the rumor, so as to convince the public and reduce the panic caused by uncertainty. Results also show that when User A clarifies the rumor based on partial information currently available, User B has a high probability of spreading rumors, and User C decides to support or oppose rumors or neutral participation according to the probability that the rumor is true and the rewards and penalties obtained from spreading rumor case. Therefore, whether User A chooses to spend time and energy on rumor information verification should also consider the positive and negative impacts of the strategies that Users B and C make. Admittedly, User A chooses to clarify rumors with verified information and can prevent User B from exploiting loopholes to maliciously spread rumors and prevent User C from spreading false information due to unknown truth. However, we can also conclude that User A’s verification strategy is limited by three factors: verifying cost, verifying period, and verification index. If the cost and time of verification exceed bounds of reason, or the mitigation index of the impact of verification information on rumors is poor, the reduction in the impact of the rumors gained using verification information may not be sufficient to motivate User A to verify the information.

### Application of results

The two models proposed in this article can be used as decision-making tools for official institutions. Firstly, the official institutions can determine rumor control strategies by considering: (1) The possible behaviors of two types of influential users in OSNs; (2) The cost of rumor clarification; (3) The impact of rumors. Secondly, publishing verified information on social media can reduce the uncertainties involved in the rumor transmission, thereby addressing the issue that the trolls use “loophole advantage” and the reputed personalities make the wrong choices due to unclear rumor information. The insights obtained from this article will be useful for official institutions to determine rumor control strategy in a rumor transmission and clarification process under different strategic conditions, which in turn will improve the rumor information dissemination and control practice during emergency events.

## Conclusion

Due to the new normal of COVID-19, people generally tend to interact online to obtain or exchange the latest information. Online social networks provide convenience for users, while the openness of social platforms also encourages false information, and rumors, that are widely spread, affecting domestic and even international public security. To avoid causing social panic, official institutions and media companies must monitor and clarify rumors. In addition to official institutions and social media companies, some unofficial individual users of social networks also actively spread rumors. They have many followers on social networks, and their comments are more influential than others. Therefore, it is important to study the interaction between the rumor control strategy of official institutions and the behavior of influential users in social networks.

Given the insufficiency of existing game theory research on the interaction between official agencies and influential users in social networks, this study creatively designs two game-theoretic models while considering the interaction behavior between official agencies (User A), trolls (User B), and reputed personalities (User C) to minimize the cost of rumor clarification and the influence of rumors in an online social network. The first model, “Rumor Clarification and Interaction Model,” serves as a decision-making tool for official rumor control agencies to make critical strategies on whether to clarify rumors by considering the cost and impact of rumors and the choice that social users will make due to the decisions of official institutions. The second model “Rumor Verification and Interaction Model” can be used to determine the best strategy for rumor control institutions to verify the information and solves the problem where trolls’ “loophole advantage” and reputed personalities make wrong choices due to unclear rumor information.

In the analysis of the two models, we determine the response boundary conditions of trolls and reputed personalities when the official institutions make different strategies and determine the subgame-perfect Nash equilibrium (SPNE) strategy of the three players. We also use numerical simulation to analyze the sensitivity of equilibrium strategies with respect to each parameter. The results of numerical analysis are helpful to determine the relative threshold that motivates players to change their strategies. Results show that the authenticity of the rumor has a strong impact on each player’s decision. When the probability of the rumor being true is low, User A chooses to clarify the rumor quickly, and User C chooses to follow User A’s judgment and publish clarification information to his followers. When the rumor information is vague, User A must verify the information first rather than clarify it quickly to avoid User B taking advantage of the loopholes to make profits, and User C should be prevented from publishing error information due to suspicion. When a rumor is likely to be true, User A can choose to ignore the rumor. At this time, User B will not choose to spread the rumor, while User C will spread the correct information to guide ordinary users. In addition, when the influence of a rumor is high, the cost of clarifying rumors is also high. User A should thus try to release verified information time and formulate a series of reward and punishment measures. Severe punishment should be used to prevent User B from spreading false information for interests; conversely, certain rewards should be set to encourage reputed personalities to participate in rumor clarification. When making decisions, User A should also consider the time and cost constraints, as well as the mitigation index of the impact of spreading correct information on rumors and the aggravation index of spreading wrong information by Users B and C. The insights gained from this study will help inform decision-makers bout the behaviors of Users A, B, and C during rumor clarification and verification in different situations, and then provide suggestions for the practice of rumor control in COVID-19.

In future work, we plan to develop more practical technologies to control the spread of rumors in OSNs. For example, influential users may be gathered on social platforms to establish a trustworthy group and provide advice to ordinary users, or a more accurate rumor identification and blocking system may be built for rumor control institutions.

## Data availability statement

The original contributions presented in this study are included in the article/Supplementary material, further inquiries can be directed to the corresponding author.

## Author contributions

WW and MJ performed the research process analysis. WW wrote the first draft of the manuscript. All authors contributed to the background, conception and design of the study, manuscript revision, read, and approved the submitted version.

## References

[B1] Agarwal P. Aziz R. A. Zhuang J. (2022). Interplay of rumor propagation and clarification on social media during crisis events – a game-theoretic approach. *Eur. J. Operat. Res.* 298, 714–733.

[B2] Ahsan M. Kumari M. Sharma T. P. (2019). Rumors detection, verification and controlling mechanisms in online social networks: A survey. *Online Soc. Netw. Med ia* 14:100050. 10.1016/j.osnem.2019.100050

[B3] Al-Garadi M. A. Varathan K. D. Ravana S. D. Ahmed E. Mujtaba G. Khan M. U. S. (2018). Analysis of online social network connections for identification of influential users: Survey and open research issues. *ACM Comput. Surv.* 51 1–37. 10.1145/3155897

[B4] Ashley Collman (2020). *Wuhan Coronavirus Outbreak.* Available online at: August, 2020 from https://www.businessinsider.com/china-information-crackdown-on-wuhan-coronavirus-C-1

[B5] Askarizadeh M. Ladani B. T. (2021). Soft rumor control in social networks: Modeling and analysis. *Eng. Appl. Artif. Intell.* 100:104198. 10.1103/PhysRevE.90.032812 25314487

[B6] Askarizadeh M. Ladani B. T. Manshaei M. H. (2019). An evolutionary game model for analysis of rumor propagation and control in social networks. *Phys. A. Stat. Mech. Appl.* 523 21–39.

[B7] Chen W. N. Tan D. Z. Yang Q. Gu T. Zhang J. (2019). Ant colony optimization for the control of pollutant spreading on social networks. *IEEE Trans. Cybern.* 50 4053–4065. 10.1109/TCYB.2019.2922266 31295135

[B8] Chua A. Y. K. Tee C. Y. Pang A. Lim E. P. (2017). The retransmission of rumor and rumor correction messages on Twitter. *Am. Behav. Sci.* 61 707–723. 10.1177/0002764217717561

[B9] Chua A. Y. Banerjee S. (2018). “Rumors and rumor corrections on Twitter: Studying message characteristics and opinion leadership,” in *Proceedings of the 2018 4th International Conference on Information Management*, (Holmdel, NJ: ICIM), 210–214. 10.1109/INFOMAN.2018.8392837

[B10] Cyberspace Administration of China (2020). *Shiyong! yiqing Piyao Chazheng Chaxun de Gongju zai Zheli [Useful! Here Come the Tools for Pandemic-Related Rumor Checking.* Beijing: Cyberspace Administration of China.

[B11] DiFonzo N. (2008). *The Watercooler Effect : A Psychologist Explores the Extraordinary Power of Rumors.* New York, NY: Penguin.

[B12] DiFonzo N. Bordia P. (2007). *Rumor Psychology: Social and organizational Approaches* (Vol. 1). Washington: American Psychological Association. 10.1037/11503-000

[B13] Flanagin A. J. Metzger M. J. (2007). The role of site features, user attributes, and information verification behaviors on the perceived credibility of web-based information. *New Media Soc.* 9 319–342. 10.1177/1461444807075015

[B14] He Z. Cai Z. Yu J. Wang X. Sun Y. Li Y. (2016). Cost-efficient strategies for restraining rumor spreading in mobile social networks. *IEEE Trans. Veh. Technol.* 66 2789–2800. 10.1109/TVT.2016.2585591

[B15] Hosni A. I. E. Li K. Ahmad S. (2020a). Analysis of the impact of online social networks addiction on the propagation of rumors. *Phys. A Stat. Mech. Appl.* 542:123456. 10.1016/j.physa.2019.123456

[B16] Hosni A. I. E. Li K. Ahmad S. (2020b). Minimizing rumor influence in multiplex online social networks based on human individual and social behaviors. *Inform. Sci.* 512 1458–1480. 10.1016/j.ins.2019.10.063

[B17] Islam A. K. M. N. Laato S. Talukder S. Sutinen E. (2020). Misinformation sharing and social media fatigue during COVID-19: An affordance and cognitive load perspective. *Technol. Forecast. Soc. Change* 159:120201. 10.1016/j.techfore.2020.120201 32834137 PMC7354273

[B18] Lee J. Y. Sundar S. S. (2013). To tweet or to retweet? That is the question for health professionals on Twitter. *Health Commun.* 28 509–524. 10.1080/10410236.2012.700391 22873787

[B19] Li Z. Zhang Q. Du X. Ma Y. Wang S. (2021). Social media rumor refutation effectiveness: Evaluation, modelling and enhancement. *Inform. Proc. Manag.* 58:102420. 10.1016/j.ipm.2020.102420

[B20] Lingam G. Rout R. R. Somayajulu D. V. L. N. (2018). Learning automata-based trust model for user recommendations in online social networks. *Comput. Electr. Eng.* 66 174–188. 10.1016/j.compeleceng.2017.10.017

[B21] Ma A. Btl A. Mhm B. (2019). An evolutionary game model for analysis of rumor propagation and control in social networks-sciencedirect. *Phys. A Stat. Mech. Appl.* 523 21–39. 10.1016/j.physa.2019.01.147

[B22] Margolin D. B. Hannak A. Weber I. (2018). Political fact-checking on Twitter: When do corrections have an effect? *Polit. Commun.* 35 196–219. 10.1080/10584609.2017.1334018

[B23] McKee M. Van Schalkwyk M. C. I. Stucker D. (2019). The second information revolution: Digital brings opportunities and concerns for public health. *Eur. J. Public Health* 29 3–6. 10.1093/eurpub/ckz160 31738440 PMC6859519

[B24] Ngamassi L. Ramakrishnan T. Rahman S. (2016). Use of social media for disaster management: A prescriptive framework. *J. Organ. End User Comput.* 28 122–140. 10.4018/JOEUC.2016070108

[B25] Pal A. Chua A. Y. Goh D. H. L. (2020). How do users respond to online rumor rebuttals? *Comput. Hum. Behav.* 106:106243. 10.1016/j.chb.2019.106243

[B26] Parimi P. Rout R. R. (2021). Genetic algorithm based rumor mitigation in online social networks through counter-rumors: A multi-objective optimization. *Inform. Proc. Manag.* 58:102669. 10.1016/j.ipm.2021.102669

[B27] Pfeffer J. Zorbach T. Carley K. M. (2014). Understanding online firestorms: Negative word-of-mouth dynamics in social media networks. *J. Mark. Commun.* 20 117–128. 10.1080/13527266.2013.797778

[B28] Prasad J. (1935). The psychology of rumour: a study relating to the great Indian earthquake of 1934. *Br. J. Psychol. Gen.* 26 1–15. 10.1111/j.2044-8295.1935.tb00770.x

[B29] Rani N. Das P. Bhardwaj A. K. (2022). Rumor, misinformation among web: A contemporary review of rumor detection techniques during different web waves. *Concurr. Comput. Pract. Exp.* 34:e6479. 10.1002/cpe.6479

[B30] Rovetta A. Bhagavathuala A. S. (2020). COVID-19-related web search behaviors and indodemic attitudes in Italy: Infodemiological study. *J. Med. Int. Res.* 6:e19274. 10.2196/19374 32338613 PMC7202310

[B31] Ruan L. Knockel J. Crete-Nishihata M. (2019). *Censored Contagion: How Information on the Coronavirus is Managed on Chinese Social Media.* The Citizen Lab. Available online at: https://citizenlab.ca/2020/03/censored-contagion-how-information-on-the-coronavirus-is-managed-on-chinese-social-media/(accessed on March 3, 2019).

[B32] Song Y. Kwon K. H. Lu Y. Fan Y. Li B. (2021). The “Parallel Pandemic” in the context of China: The spread of rumors and rumor-corrections During COVID-19 in Chinese social media. *Am. Behav. Sci.* 65 2014–2036. 10.1177/00027642211003153 PMC7992098 38603026

[B33] Srinivasan S. Ld D. B. (2021b). A social immunity based approach to suppress rumors in online social networks. *Intl. J. Mach. Learn. Cybern.* 12 1281–1296. 10.1007/s13042-020-01233-0

[B34] Srinivasan S. Ld D. B. (2021a). A Bio-inspired defensive rumor confinement strategy in online social networks. *J. Organ. End User Comput.* 33 47–70. 10.4018/JOEUC.2021010103

[B35] Subramaniyaswamy V. Logesh R. Abejith M. Umasankar S. Umamakeswari A. (2017). Sentiment analysis of tweets for estimating criticality and security of event. *J. Organ. End User Comput.* 29 51–71. 10.4018/JOEUC.2017100103

[B36] Vosoughi S. Roy D. Aral S. (2018). The spread of true and false news online. *Science* 359 1146–1151. 10.1126/science.aap9559 29590045

[B37] Wang B. Zhuang J. (2018). Rumor response, debunking response, and decision makings of misinformed Twitter users during disaster. *Nat. Hazards* 93 1145–1162. 10.1007/s11069-018-3344-6

[B38] Wang J. Xie Z. Li Q. Tan J. Xing R. Chen Y. (2019). Effect of digitalized rumor clarification on stock markets. *Emerg. Mark. Finance Trade* 55 450–474. 10.1080/1540496X.2018.1534683

[B39] Wang X. Li Y. Li J. Liu Y. Qiu C. (2021). A rumor reversal model of online health information during the Covid-19 epidemic. *Inform. Proc. Manag.* 58:102731. 10.1016/j.ipm.2021.102731 34539040 PMC8441309

[B40] Wang Y. Zheng L. Zuo J. (2021). Online rumor propagation of social media on NIMBY conflict: Temporal patterns, frameworks and rumor-mongers. *Environ. Impact Assess. Rev.* 91:106647. 10.1016/j.eiar.2021.106647

[B41] Wang Z. Guo Y. (2020). Empower rumor events detection from Chinese microblogs with multi-type individual information. *Knowl. Inform. Syst.* 62 3585–3614. 10.1007/s10115-020-01463-2

[B42] Weeks B. E. Garrett R. K. (2014). Electoral consequences of political rumors: Motivated reasoning, candidate rumors, and vote choice during the 2008 US presidential election. *Intl. J. Public Opin. Res.* 26 401–422. 10.1093/ijpor/edu005

[B43] Weibo Piyao (2020). *Weibo Piyao Yuedu Gongzuo Baogao (2021 nian 9 yue) [Weibo Rumor Rebuttal Monthly Report (2021, September)].* Sina Weibo. Available online at: https://weibo.com/ttarticle/p/show?id=2309404692506657816701#_0 (accessed on October 16, 2020).

[B44] Wen S. Jiang J. Xiang Y. Yu S. Zhou W. Jia W. (2014). To shut them up or to clarify: Restraining the spread of rumors in online social networks. *IEEE Trans. Parallel Distrib. Syst*. 25, 3306–3316.

[B45] Xiao Y. Chen D. Wei S. Li Q. Wang H. Xu M. (2019). Rumor propagation dynamic model based on evolutionary game and anti-rumor. *Nonlinear Dyn.* 95 523–539. 10.1007/s11071-018-4579-1

[B46] Yan R. Li D. Wu W. Du D. Z. Wang Y. (2019). Minimizing influence of rumors by blockers on social networks: algorithms and analysis. *IEEE Trans. Netw. Sci. Eng.* 7 1067–1078. 10.1109/TNSE.2019.2903272

[B47] Yang L. Li Z. Giua A. (2020). Containment of rumor spread in complex social networks. *Inform. Sci.* 506 113–130. 10.1371/journal.pone.0229201 32163423 PMC7067483

[B48] Yang Q. Tufts C. Ungar L. Guntuku S. Merchant R. (2018). To retweet or not to retweet: Understanding what features of cardiovascular tweets influence their retransmission. *J. Health Commun.* 23 1026–1035. 10.1080/10810730.2018.1540671 30404564 PMC6463511

[B49] Yao X. Gu Y. Gu C. Huang H. (2022). Fast controlling of rumors with limited cost in social networks. *Comput. Commun.* 182 41–51.

[B50] Yao X. Liang G. Gu C. Huang H. (2021). Rumors clarification with minimum credibility in social networks. *Comput. Netw.* 193:108123. 10.1016/j.comnet.2021.108123 PMC9760356 36567704

[B51] Zareie A. Sakellariou R. (2021). Minimizing the spread of misinformation in online social networks: A survey. *J. Netw. Comput. Appl.* 186:103094. 10.1016/j.cct.2022.106779 35491009

[B52] Zhang X. Zhu H. Hwang Y. Xiao C. (2022). Sharing or Not: Psychological motivations of brand rumors spread and the stop solutions. *Front. Psychol.* 13:830002. 10.3389/fpsyg.2022.830002 35444586 PMC9015072

[B53] Zhuang Y. B. Chen J. J. Li Z. H. (2017). Modeling the cooperative and competitive contagions in online social networks. *Physica A Stat. Mech. Appl.* 484 141–151. 10.1016/j.physa.2017.04.129

[B54] Zhuang Y. B. Li Z. H. Zhuang Y. J. (2021). Identification of influencers in online social networks: measuring influence considering multidimensional factors exploration. *Heliyon* 7:e06472. 10.1016/j.heliyon.2021.e06472 33898799 PMC8060605

[B55] Zubiaga A. Liakata M. Procter R. Hoi G. Tolmie P. (2016). Analysing how people orient to and spread rumours in social media by looking at conversational threads. *PLoS One* 11:e0150989. 10.1371/journal.pone.0150989 26943909 PMC4778911

