# Supplementary material for: Interaction between official institutions and influential users of rumor control in online social networks
Source: Front Psychol. 2022 Aug 2;13:937296. doi: 10.3389/fpsyg.2022.937296 (PMC9379133; doi:10.3389/fpsyg.2022.937296)
Supplement: Supplementary file 1 [file Data_Sheet_1.docx]

**Appendix A**

A1.Proof of Proposition 1

When$n=1$, User A choose to clarify the rumor. Expected profit of User B while propagating rumor,$M_{1B}(y_{1}=P)=(a_{1}+r_{1}^{S}+r_{1}^{P})f_{1}+pe-(1-p)p_{d}c_{1}$, Expected profit of User B while terminating propagate rumor, $M_{1B}(y_{1}=T)=0$.

For$\hat{y}_{n}\equiv P$, the following condition must hold: $M_{1B}(y_{1}=P)\geq M_{1B}(y_{1}=T)$, by solving the inequality, we can obtain the following condition: $p_{d}\leq\frac{(a_{1}+r_{1}^{S}+r_{1}^{P})f_{1}+pe}{(1-p)c_{1}}$ .

For$\hat{y}_{n}\equiv T$, the following condition must hold: $M_{1B}(y_{1}=T)\geq M_{1B}(y_{1}=P)$, by solving the inequality, we can obtain the following condition: $p_{d}\geq\frac{(a_{1}+r_{1}^{S}+r_{1}^{P})f_{1}+pe}{(1-p)c_{1}}$ .

When$n=2$, User A choose to disregard the rumor. Expected profit of User B while propagating rumor,$M_{1B}(y_{2}=P)=(a_{1}+r_{1}^{S}+r_{1}^{P})f_{1}$, Expected profit of User B while terminating propagate rumor, $M_{1B}(y_{1}=T)=0$. The strategy P always takes precedence over T, therefore, $\hat{y}_{n}\equiv P$.

A2. Proof of Proposition 2

When$n=1$, User A choose to clarify the rumor. Expected utility of User C while disseminating the clarified information,$U_{1C}(z_{1}=Q)=(a_{2}+r_{2}^{S}+r_{2}^{P})f_{2}+e$. Expected utility of User C while neutral participating regarding the clarification,$U_{1C}(z_{1}=N)=0$. The strategy Q always takes precedence over N, therefore, $\hat{z}_{n}\equiv Q$

When$n=2$, User A choose to disregard the rumor. Expected utility of User C while supporting the rumor, $U_{1C}(z_{2}=S)=(pa_{2}-(1-p)c_{2}+r_{2}^{S}+r_{2}^{P})f_{2}$. Expected utility of User C while opposing the rumor, $U_{1C}(z_{2}=K)=((1-p)a_{2}-pc_{2}+r_{2}^{S}+r_{2}^{P})f_{2}$. Expected utility of User C while neutral participating regarding the clarification,$U_{1C}(z_{2}=N)=0$.

For$\hat{z}_{n}\equiv S$, the following condition must hold: $\left\{\begin{matrix} U_{1C}(z_{2}=S)\geq U_{1C}(z_{2}=K) \\ U_{1C}(z_{2}=S)\geq U_{1C}(z_{2}=N) \end{matrix}\right.$, by solving the inequality, we can obtain the following condition:$p\geq\max(\frac{1}{2},\frac{c_{2}-(r_{2}^{S}+r_{2}^{P})}{a_{2}+c_{2}})$.

For$\hat{z}_{n}\equiv K$, the following condition must hold: $\left\{\begin{matrix} U_{1C}(z_{2}=K)\geq U_{1C}(z_{2}=S) \\ U_{1C}(z_{2}=K)\geq U_{1C}(z_{2}=N) \end{matrix}\right.$, by solving the inequality, we can obtain the following condition:$p\leq\min(\frac{1}{2},\frac{a_{2}+(r_{2}^{S}+r_{2}^{P})}{a_{2}+c_{2}})$.

For$\hat{z}_{n}\equiv N$, the following condition must hold: $\left\{\begin{matrix} U_{1C}(z_{2}=K)\geq U_{1C}(z_{2}=S) \\ U_{1C}(z_{2}=K)\geq U_{1C}(z_{2}=N) \end{matrix}\right.$, by solving the inequality, we can obtain the following condition:$p\in(\frac{a_{2}+(r_{2}^{S}+r_{2}^{P})}{a_{2}+c_{2}},\frac{c_{2}-(r_{2}^{S}+r_{2}^{P})}{a_{2}+c_{2}})$.

A3. Proof of Proposition 3

Taking the best response functions of User B and user C defined in [Eq.(4)](#Equi4) and [Eq.(5)](#Equi5) into the expected loss function [Eq.(1)](#Equi1) to obtain the following expressions for$L_{1A}$in terms of $\hat{y}_{n}$ and $\hat{z}_{n}$:

$L_{1A}(x=D,\hat{y}_{n}=P,\hat{z}_{n}=Q)=r^{D}+(pu_{1}+(1-p)v_{1})u_{2}dr^{H}$, subject to $C_{1}\equiv p\geq\frac{p_{d}c_{1}-(a_{1}+r_{1}^{S}+r_{1}^{P})f_{1}}{e+p_{d}c_{1}}$;

$L_{1A}(x=D,\hat{y}_{n}=T,\hat{z}_{n}=Q)=r^{D}+u_{2}dr^{H}$, subject to $C_{2}\equiv p\leq\frac{p_{d}c_{1}-(a_{1}+r_{1}^{S}+r_{1}^{P})f_{1}}{e+p_{d}c_{1}}$;

$L_{1A}(x=ND,\hat{y}_{n}=P,\hat{z}_{n}=S)=(pu_{1}+(1-p)v_{1})\cdot(pu_{2}+(1-p)v_{2})r^{H}$, subject to $C_{3}\equiv p\geq\max(\frac{1}{2},\frac{c_{2}-(r_{2}^{S}+r_{2}^{P})}{a_{2}+c_{2}})$;

$L_{1A}(x=ND,\hat{y}_{n}=P,\hat{z}_{n}=K)=(pu_{1}+(1-p)v_{1})\cdot(pv_{2}+(1-p)u_{2})r^{H}$, subject to $C_{4}\equiv p\leq\min(\frac{1}{2},\frac{a_{2}+(r_{2}^{S}+r_{2}^{P})}{a_{2}+c_{2}})$;

$L_{1A}(x=ND,\hat{y}_{n}=P,\hat{z}_{n}=N)=r^{H}$, subject to $C_{5}\equiv p\in(\frac{a_{2}+(r_{2}^{S}+r_{2}^{P})}{a_{2}+c_{2}},\frac{c_{2}-(r_{2}^{S}+r_{2}^{P})}{a_{2}+c_{2}})$.

For $(x^{*},y^{*},z^{*})=(D,P,Q)$, The following conditions must be met:

$$\begin{matrix} R_{1}\equiv\{\{L_{1A}(x=D,\hat{y}_{n}=P,\hat{z}_{n}=Q)\leq L_{1A}(x=D,\hat{y}_{n}=T,\hat{z}_{n}=Q)\cap(C_{1}\cap C_{2})\} \\ \text{ }\cap\{L_{1A}(x=D,\hat{y}_{n}=P,\hat{z}_{n}=Q)\leq L_{1A}(x=ND,\hat{y}_{n}=P,\hat{z}_{n}=S)\cap(C_{1}\cap C_{3})\} \\ \text{ }\cup\{L_{1A}(x=D,\hat{y}_{n}=P,\hat{z}_{n}=Q)\leq L_{1A}(x=ND,\hat{y}_{n}=P,\hat{z}_{n}=K)\cap(C_{1}\cap C_{4})\} \\ \text{ }\cup\{L_{1A}(x=D,\hat{y}_{n}=P,\hat{z}_{n}=Q)\leq L_{1A}(x=ND,\hat{y}_{n}=P,\hat{z}_{n}=N)\cap(C_{1}\cap C_{5})\}\} \end{matrix}$$

For $(x^{*},y^{*},z^{*})=(D,T,Q)$, The following conditions must be met:

$$\begin{matrix} R_{2}\equiv\{\{L_{1A}(x=D,\hat{y}_{n}=T,\hat{z}_{n}=Q)\leq L_{1A}(x=D,\hat{y}_{n}=P,\hat{z}_{n}=Q)\cap(C_{2}\cap C_{1})\} \\ \text{ }\cap\{L_{1A}(x=D,\hat{y}_{n}=T,\hat{z}_{n}=Q)\leq L_{1A}(x=ND,\hat{y}_{n}=P,\hat{z}_{n}=S)\cap(C_{2}\cap C_{3})\} \\ \text{ }\cup\{L_{1A}(x=D,\hat{y}_{n}=T,\hat{z}_{n}=Q)\leq L_{1A}(x=ND,\hat{y}_{n}=P,\hat{z}_{n}=K)\cap(C_{2}\cap C_{4})\} \\ \text{ }\cup\{L_{1A}(x=D,\hat{y}_{n}=T,\hat{z}_{n}=Q)\leq L_{1A}(x=ND,\hat{y}_{n}=P,\hat{z}_{n}=N)\cap(C_{2}\cap C_{5})\}\} \end{matrix}$$

For $(x^{*},y^{*},z^{*})=(ND,P,S)$, The following conditions must be met:

$$\begin{matrix} R_{3}\equiv\{\{L_{1A}(x=ND,\hat{y}_{n}=P,\hat{z}_{n}=S)\leq L_{1A}(x=D,\hat{y}_{n}=P,\hat{z}_{n}=Q)\cap(C_{3}\cap C_{1})\} \\ \text{ }\cup\{L_{1A}(x=ND,\hat{y}_{n}=P,\hat{z}_{n}=S)\leq L_{1A}(x=D,\hat{y}_{n}=T,\hat{z}_{n}=Q)\cap(C_{3}\cap C_{2})\} \\ \text{ }\cap\{L_{1A}(x=ND,\hat{y}_{n}=P,\hat{z}_{n}=S)\leq L_{1A}(x=ND,\hat{y}_{n}=P,\hat{z}_{n}=K)\cap(C_{3}\cap C_{4})\} \\ \text{ }\cup\{L_{1A}(x=ND,\hat{y}_{n}=P,\hat{z}_{n}=S)\leq L_{1A}(x=ND,\hat{y}_{n}=P,\hat{z}_{n}=N)\cap(C_{3}\cap C_{5})\}\} \end{matrix}$$

For $(x^{*},y^{*},z^{*})=(ND,P,K)$, The following conditions must be met:

$$\begin{matrix} R_{4}\equiv\{\{L_{1A}(x=ND,\hat{y}_{n}=P,\hat{z}_{n}=K)\leq L_{1A}(x=D,\hat{y}_{n}=P,\hat{z}_{n}=Q)\cap(C_{4}\cap C_{1})\} \\ \text{ }\cup\{L_{1A}(x=ND,\hat{y}_{n}=P,\hat{z}_{n}=K)\leq L_{1A}(x=D,\hat{y}_{n}=T,\hat{z}_{n}=Q)\cap(C_{4}\cap C_{2})\} \\ \text{ }\cap\{L_{1A}(x=ND,\hat{y}_{n}=P,\hat{z}_{n}=K)\leq L_{1A}(x=ND,\hat{y}_{n}=P,\hat{z}_{n}=S)\cap(C_{4}\cap C_{3})\} \\ \text{ }\cup\{L_{1A}(x=ND,\hat{y}_{n}=P,\hat{z}_{n}=K)\leq L_{1A}(x=ND,\hat{y}_{n}=P,\hat{z}_{n}=N)\cap(C_{4}\cap C_{5})\}\} \end{matrix}$$

For $(x^{*},y^{*},z^{*})=(ND,P,N)$, The following conditions must be met:

$$\begin{matrix} R_{5}\equiv\{\{L_{1A}(x=ND,\hat{y}_{n}=P,\hat{z}_{n}=N)\leq L_{1A}(x=D,\hat{y}_{n}=P,\hat{z}_{n}=Q)\cap(C_{5}\cap C_{1})\} \\ \text{ }\cup\{L_{1A}(x=ND,\hat{y}_{n}=P,\hat{z}_{n}=N)\leq L_{1A}(x=D,\hat{y}_{n}=T,\hat{z}_{n}=Q)\cap(C_{5}\cap C_{2})\} \\ \text{ }\cap\{L_{1A}(x=ND,\hat{y}_{n}=P,\hat{z}_{n}=N)\leq L_{1A}(x=ND,\hat{y}_{n}=P,\hat{z}_{n}=S)\cap(C_{5}\cap C_{3})\} \\ \text{ }\cap\{L_{1A}(x=ND,\hat{y}_{n}=P,\hat{z}_{n}=N)\leq L_{1A}(x=ND,\hat{y}_{n}=P,\hat{z}_{n}=K)\cap(C_{5}\cap C_{4})\}\} \end{matrix}$$

A4. Proof of Proposition 4

When$n=1$, User A choose to clarify the rumor. Expected profit of User B while propagating rumor,$M_{2B}(y_{1}=P)=(a_{1}+r_{1}^{S}+r_{1}^{P})f_{1}+pe-(1-p)p_{d}c_{1}$, Expected profit of User B while terminating propagate rumor, $M_{2B}(y_{1}=T)=0$.

For$\hat{y}_{n}\equiv P$, the following condition must hold: $M_{2B}(y_{1}=P)\geq M_{2B}(y_{1}=T)$, by solving the inequality, we can obtain the following condition:$p_{d}\leq\frac{(a_{1}+r_{1}^{S}+r_{1}^{P})f_{1}+pe}{(1-p)c_{1}}$ .

For$\hat{y}_{n}\equiv T$, the following condition must hold: $M_{2B}(y_{1}=T)\geq M_{2B}(y_{1}=P)$, by solving the inequality, we can obtain the following condition: $p_{d}\geq\frac{(a_{1}+r_{1}^{S}+r_{1}^{P})f_{1}+pe}{(1-p)c_{1}}$ .

When$n=2$, User A choose to disregard the rumor. Expected profit of User B while propagating rumor,$M_{1B}(y_{2}=P)=(a_{1}+r_{1}^{S}+r_{1}^{P})f_{1}-c_{1}p_{d}$, Expected profit of User B while terminating propagate rumor, $M_{2B}(y_{2}=T)=0$.

For$\hat{y}_{n}\equiv P$, the following condition must hold: $M_{2B}(y_{2}=P)\geq M_{2B}(y_{2}=T)$, by solving the inequality, we can obtain the following condition:$p_{d}\leq\frac{(a_{1}+r_{1}^{S}+r_{1}^{P})f_{1}}{c_{1}}$ .

For$\hat{y}_{n}\equiv T$, the following condition must hold: $M_{2B}(y_{2}=T)\geq M_{2B}(y_{2}=P)$, by solving the inequality, we can obtain the following condition: $p_{d}\geq\frac{(a_{1}+r_{1}^{S}+r_{1}^{P})f_{1}}{c_{1}}$ .

A5. Proof of Proposition 5

When$n=1$, User A choose to disregard the rumor. Expected utility of User C while supporting the rumor, $U_{2C}(z_{1}=S)=(pa_{2}-(1-p)c_{2}+r_{2}^{S}+r_{2}^{P})f_{2}+pe$. Expected utility of User C while opposing the rumor, $U_{2C}(z_{1}=K)=((1-p)a_{2}-pc_{2}+r_{2}^{S}+r_{2}^{P})f_{2}+(1-p)e$. Expected utility of User C while neutral participating regarding the clarification,$U_{2C}(z_{1}=N)=0$.

For$\hat{z}_{n}\equiv S$, the following condition must hold: $\left\{\begin{matrix} U_{2C}(z_{1}=S)\geq U_{2C}(z_{1}=K) \\ U_{2C}(z_{1}=S)\geq U_{2C}(z_{1}=N) \end{matrix}\right.$, by solving the inequality, we can obtain the following condition:$p\geq\max(\frac{1}{2},\frac{(c_{2}-r_{2}^{S}-r_{2}^{P})f_{2}}{(a_{2}+c_{2})f_{2}+e})$.

For$\hat{z}_{n}\equiv K$, the following condition must hold: $\left\{\begin{matrix} U_{2C}(z_{1}=K)\geq U_{2C}(z_{1}=S) \\ U_{2C}(z_{1}=K)\geq U_{2C}(z_{1}=N) \end{matrix}\right.$, by solving the inequality, we can obtain the following condition:$p\leq\min(\frac{1}{2},\frac{(a_{2}+r_{2}^{S}+r_{2}^{P})f_{2}+e}{(a_{2}+c_{2})f_{2}+e})$.

For$\hat{z}_{n}\equiv N$, the following condition must hold: $\left\{\begin{matrix} U_{2C}(z_{1}=K)\geq U_{2C}(z_{1}=S) \\ U_{2C}(z_{1}=K)\geq U_{2C}(z_{1}=N) \end{matrix}\right.$, by solving the inequality, we can obtain the following condition:$p\in(\frac{(a_{2}+r_{2}^{S}+r_{2}^{P})f_{2}+e}{(a_{2}+c_{2})f_{2}+e},\frac{(c_{2}-r_{2}^{S}-r_{2}^{P})f_{2}}{(a_{2}+c_{2})f_{2}+e})$.

When$n=2$, User A choose to clarify the rumor. Expected utility of User C while disseminating the clarified information,$U_{2C}(z_{2}=Q)=(a_{2}+r_{2}^{S}+r_{2}^{P})f_{2}+e$. Expected utility of User C while neutral participating regarding the clarification,$U_{2C}(z_{2}=N)=0$. The strategy Q always takes precedence over N, therefore, $\hat{z}_{n}\equiv Q$

A6. Proof of Proposition 6

Taking the best response functions of User B and user C defined in [Eq.(14)](#Equi14) and [Eq.(15)](#Equi15) into the expected loss function [Eq.(11)](#Equi11) to obtain the following expressions for$L_{2A}$in terms of $\hat{y}_{n}$ and $\hat{z}_{n}$:

$L_{2A}(x=D,\hat{y}_{n}=P,\hat{z}_{n}=S)=r^{D}+(pu_{1}+(1-p)v_{1})(pu_{2}+(1-p)v_{2})dr^{H}$, subject to $F_{1}\equiv\left\{\begin{matrix} p_{d}\leq\frac{(a_{1}+r_{1}^{S}+r_{1}^{P})f_{1}+pe}{(1-p)c_{1}} \\ p\geq\max(\frac{1}{2},\frac{(c_{2}-r_{2}^{S}-r_{2}^{P})f_{2}}{(a_{2}+c_{2})f_{2}+e}) \end{matrix}\right.$;

$L_{2A}(x=D,\hat{y}_{n}=P,\hat{z}_{n}=K)=r^{D}+(pu_{1}+(1-p)v_{1})\cdot(pv_{2}+(1-p)u_{2})dr^{H}$, subject to$F_{2}\equiv\left\{\begin{matrix} p_{d}\leq\frac{(a_{1}+r_{1}^{S}+r_{1}^{P})f_{1}+pe}{(1-p)c_{1}} \\ p\leq\min(\frac{1}{2},\frac{(a_{2}+r_{2}^{S}+r_{2}^{P})f_{2}+e}{(a_{2}+c_{2})f_{2}+e}) \end{matrix}\right.$;

$L_{2A}(x=D,\hat{y}_{n}=P,\hat{z}_{n}=N)=r^{D}+(pu_{1}+(1-p)v_{1})dr^{H}$, subject to$F_{3}\equiv\left\{\begin{matrix} p_{d}\leq\frac{(a_{1}+r_{1}^{S}+r_{1}^{P})f_{1}+pe}{(1-p)c_{1}} \\ p\in(\frac{(a_{2}+r_{2}^{S}+r_{2}^{P})f_{2}+e}{(a_{2}+c_{2})f_{2}+e},\frac{(c_{2}-r_{2}^{S}-r_{2}^{P})f_{2}}{(a_{2}+c_{2})f_{2}+e}) \end{matrix}\right.$;

$L_{2A}(x=D,\hat{y}_{n}=T,\hat{z}_{n}=S)=r^{D}+(pu_{2}+(1-p)v_{2})dr^{H}$, subject to$F_{4}\equiv\left\{\begin{matrix} p_{d}\geq\frac{(a_{1}+r_{1}^{S}+r_{1}^{P})f_{1}+pe}{(1-p)c_{1}} \\ p\geq\max(\frac{1}{2},\frac{(c_{2}-r_{2}^{S}-r_{2}^{P})f_{2}}{(a_{2}+c_{2})f_{2}+e}) \end{matrix}\right.$;

$L_{2A}(x=D,\hat{y}_{n}=T,\hat{z}_{n}=K)=r^{D}+(pv_{2}+(1-p)u_{2})dr^{H}$, subject to$F_{5}\equiv\left\{\begin{matrix} p_{d}\geq\frac{(a_{1}+r_{1}^{S}+r_{1}^{P})f_{1}+pe}{(1-p)c_{1}} \\ p\leq\min(\frac{1}{2},\frac{(a_{2}+r_{2}^{S}+r_{2}^{P})f_{2}+e}{(a_{2}+c_{2})f_{2}+e}) \end{matrix}\right.$.

$L_{2A}(x=D,\hat{y}_{n}=T,\hat{z}_{n}=N)=r^{D}+dr^{H}$, subject to$F_{6}\equiv\left\{\begin{matrix} p_{d}\geq\frac{(a_{1}+r_{1}^{S}+r_{1}^{P})f_{1}+pe}{(1-p)c_{1}} \\ p\in(\frac{(a_{2}+r_{2}^{S}+r_{2}^{P})f_{2}+e}{(a_{2}+c_{2})f_{2}+e},\frac{(c_{2}-r_{2}^{S}-r_{2}^{P})f_{2}}{(a_{2}+c_{2})f_{2}+e}) \end{matrix}\right.$.

$L_{2A}(x=VD,\hat{y}_{n}=P,\hat{z}_{n}=Q)=r^{D}+r^{V}t+v_{1}u_{2}dlr^{H}$, subject to$F_{7}\equiv p_{d}\leq\frac{(a_{1}+r_{1}^{S}+r_{1}^{P})f_{1}}{c_{1}}$.

$L_{2A}(x=VD,\hat{y}_{n}=T,\hat{z}_{n}=Q)=r^{D}+r^{V}t+u_{2}dlr^{H}$, subject to$F_{8}\equiv p_{d}\geq\frac{(a_{1}+r_{1}^{S}+r_{1}^{P})f_{1}}{c_{1}}$.

For $(x^{*},y^{*},z^{*})=(D,P,S)$, The following conditions must be met:

$$\begin{matrix} V_{1}\equiv\{\{L_{2A}(x=D,\hat{y}_{n}=P,\hat{z}_{n}=S)\leq L_{2A}(x=D,\hat{y}_{n}=P,\hat{z}_{n}=K)\cap(F_{1}\cap F_{2})\} \\ \text{ }\cup\{L_{2A}(x=D,\hat{y}_{n}=P,\hat{z}_{n}=S)\leq L_{2A}(x=D,\hat{y}_{n}=P,\hat{z}_{n}=N)\cap(F_{1}\cap F_{3})\} \\ \text{ }\cap\{L_{2A}(x=D,\hat{y}_{n}=P,\hat{z}_{n}=S)\leq L_{2A}(x=D,\hat{y}_{n}=T,\hat{z}_{n}=S)\cap(F_{1}\cap F_{4})\} \\ \text{ }\cup\{L_{2A}(x=D,\hat{y}_{n}=P,\hat{z}_{n}=S)\leq L_{2A}(x=D,\hat{y}_{n}=T,\hat{z}_{n}=K)\cap(F_{1}\cap F_{5})\} \\ \text{ }\cup\{L_{2A}(x=D,\hat{y}_{n}=P,\hat{z}_{n}=S)\leq L_{2A}(x=D,\hat{y}_{n}=T,\hat{z}_{n}=N)\cap(F_{1}\cap F_{6})\} \\ \text{ }\cap\{L_{2A}(x=D,\hat{y}_{n}=P,\hat{z}_{n}=S)\leq L_{2A}(x=VD,\hat{y}_{n}=P,\hat{z}_{n}=Q)\cap(F_{1}\cap F_{7})\} \\ \text{ }\cup\{L_{2A}(x=D,\hat{y}_{n}=P,\hat{z}_{n}=S)\leq L_{2A}(x=VD,\hat{y}_{n}=T,\hat{z}_{n}=Q)\cap(F_{1}\cap F_{8})\}\} \end{matrix}$$

For $(x^{*},y^{*},z^{*})=(D,P,K)$, The following conditions must be met:

$$\begin{matrix} V_{2}\equiv\{\{L_{2A}(x=D,\hat{y}_{n}=P,\hat{z}_{n}=K)\leq L_{2A}(x=D,\hat{y}_{n}=P,\hat{z}_{n}=S)\cap(F_{2}\cap F_{1})\} \\ \text{ }\cup\{L_{2A}(x=D,\hat{y}_{n}=P,\hat{z}_{n}=K)\leq L_{2A}(x=D,\hat{y}_{n}=P,\hat{z}_{n}=N)\cap(F_{2}\cap F_{3})\} \\ \text{ }\cap\{L_{2A}(x=D,\hat{y}_{n}=P,\hat{z}_{n}=K)\leq L_{2A}(x=D,\hat{y}_{n}=T,\hat{z}_{n}=S)\cap(F_{2}\cap F_{4})\} \\ \text{ }\cup\{L_{2A}(x=D,\hat{y}_{n}=P,\hat{z}_{n}=K)\leq L_{2A}(x=D,\hat{y}_{n}=T,\hat{z}_{n}=K)\cap(F_{2}\cap F_{5})\} \\ \text{ }\cup\{L_{2A}(x=D,\hat{y}_{n}=P,\hat{z}_{n}=K)\leq L_{2A}(x=D,\hat{y}_{n}=T,\hat{z}_{n}=N)\cap(F_{2}\cap F_{6})\} \\ \text{ }\cap\{L_{2A}(x=D,\hat{y}_{n}=P,\hat{z}_{n}=K)\leq L_{2A}(x=VD,\hat{y}_{n}=P,\hat{z}_{n}=Q)\cap(F_{2}\cap F_{7})\} \\ \text{ }\cup\{L_{2A}(x=D,\hat{y}_{n}=P,\hat{z}_{n}=K)\leq L_{2A}(x=VD,\hat{y}_{n}=T,\hat{z}_{n}=Q)\cap(F_{2}\cap F_{8})\}\} \end{matrix}$$

For $(x^{*},y^{*},z^{*})=(D,P,N)$, The following conditions must be met:

$$\begin{matrix} V_{3}\equiv\{\{L_{2A}(x=D,\hat{y}_{n}=P,\hat{z}_{n}=N)\leq L_{2A}(x=D,\hat{y}_{n}=P,\hat{z}_{n}=S)\cap(F_{3}\cap F_{1})\} \\ \text{ }\cup\{L_{2A}(x=D,\hat{y}_{n}=P,\hat{z}_{n}=N)\leq L_{2A}(x=D,\hat{y}_{n}=P,\hat{z}_{n}=K)\cap(F_{3}\cap F_{2})\} \\ \text{ }\cap\{L_{2A}(x=D,\hat{y}_{n}=P,\hat{z}_{n}=N)\leq L_{2A}(x=D,\hat{y}_{n}=T,\hat{z}_{n}=S)\cap(F_{3}\cap F_{4})\} \\ \text{ }\cup\{L_{2A}(x=D,\hat{y}_{n}=P,\hat{z}_{n}=N)\leq L_{2A}(x=D,\hat{y}_{n}=T,\hat{z}_{n}=K)\cap(F_{3}\cap F_{5})\} \\ \text{ }\cup\{L_{2A}(x=D,\hat{y}_{n}=P,\hat{z}_{n}=N)\leq L_{2A}(x=D,\hat{y}_{n}=T,\hat{z}_{n}=N)\cap(F_{3}\cap F_{6})\} \\ \text{ }\cap\{L_{2A}(x=D,\hat{y}_{n}=P,\hat{z}_{n}=N)\leq L_{2A}(x=VD,\hat{y}_{n}=P,\hat{z}_{n}=Q)\cap(F_{3}\cap F_{7})\} \\ \text{ }\cup\{L_{2A}(x=D,\hat{y}_{n}=P,\hat{z}_{n}=N)\leq L_{2A}(x=VD,\hat{y}_{n}=T,\hat{z}_{n}=Q)\cap(F_{3}\cap F_{8})\}\} \end{matrix}$$

For $(x^{*},y^{*},z^{*})=(D,T,S)$, The following conditions must be met:

$$\begin{matrix} V_{4}\equiv\{\{L_{2A}(x=D,\hat{y}_{n}=T,\hat{z}_{n}=S)\leq L_{2A}(x=D,\hat{y}_{n}=P,\hat{z}_{n}=S)\cap(F_{4}\cap F_{1})\} \\ \text{ }\cup\{L_{2A}(x=D,\hat{y}_{n}=T,\hat{z}_{n}=S)\leq L_{2A}(x=D,\hat{y}_{n}=P,\hat{z}_{n}=K)\cap(F_{4}\cap F_{2})\} \\ \text{ }\cup\{L_{2A}(x=D,\hat{y}_{n}=T,\hat{z}_{n}=S)\leq L_{2A}(x=D,\hat{y}_{n}=P,\hat{z}_{n}=N)\cap(F_{4}\cap F_{3})\} \\ \text{ }\cap\{L_{2A}(x=D,\hat{y}_{n}=T,\hat{z}_{n}=S)\leq L_{2A}(x=D,\hat{y}_{n}=T,\hat{z}_{n}=K)\cap(F_{4}\cap F_{5})\} \\ \text{ }\cup\{L_{2A}(x=D,\hat{y}_{n}=T,\hat{z}_{n}=S)\leq L_{2A}(x=D,\hat{y}_{n}=T,\hat{z}_{n}=N)\cap(F_{4}\cap F_{6})\} \\ \text{ }\cap\{L_{2A}(x=D,\hat{y}_{n}=T,\hat{z}_{n}=S)\leq L_{2A}(x=VD,\hat{y}_{n}=P,\hat{z}_{n}=Q)\cap(F_{4}\cap F_{7})\} \\ \text{ }\cup\{L_{2A}(x=D,\hat{y}_{n}=T,\hat{z}_{n}=S)\leq L_{2A}(x=VD,\hat{y}_{n}=T,\hat{z}_{n}=Q)\cap(F_{4}\cap F_{8})\}\} \end{matrix}$$

For $(x^{*},y^{*},z^{*})=(D,T,K)$, The following conditions must be met:

$$\begin{matrix} V_{5}\equiv\{\{L_{2A}(x=D,\hat{y}_{n}=T,\hat{z}_{n}=K)\leq L_{2A}(x=D,\hat{y}_{n}=P,\hat{z}_{n}=S)\cap(F_{5}\cap F_{1})\} \\ \text{ }\cup\{L_{2A}(x=D,\hat{y}_{n}=T,\hat{z}_{n}=K)\leq L_{2A}(x=D,\hat{y}_{n}=P,\hat{z}_{n}=K)\cap(F_{5}\cap F_{2})\} \\ \text{ }\cup\{L_{2A}(x=D,\hat{y}_{n}=T,\hat{z}_{n}=K)\leq L_{2A}(x=D,\hat{y}_{n}=P,\hat{z}_{n}=N)\cap(F_{5}\cap F_{3})\} \\ \text{ }\cap\{L_{2A}(x=D,\hat{y}_{n}=T,\hat{z}_{n}=K)\leq L_{2A}(x=D,\hat{y}_{n}=T,\hat{z}_{n}=S)\cap(F_{5}\cap F_{4})\} \\ \text{ }\cup\{L_{2A}(x=D,\hat{y}_{n}=T,\hat{z}_{n}=K)\leq L_{2A}(x=D,\hat{y}_{n}=T,\hat{z}_{n}=N)\cap(F_{5}\cap F_{6})\} \\ \text{ }\cap\{L_{2A}(x=D,\hat{y}_{n}=T,\hat{z}_{n}=K)\leq L_{2A}(x=VD,\hat{y}_{n}=P,\hat{z}_{n}=Q)\cap(F_{5}\cap F_{7})\} \\ \text{ }\cup\{L_{2A}(x=D,\hat{y}_{n}=T,\hat{z}_{n}=K)\leq L_{2A}(x=VD,\hat{y}_{n}=T,\hat{z}_{n}=Q)\cap(F_{5}\cap F_{8})\}\} \end{matrix}$$

For $(x^{*},y^{*},z^{*})=(D,T,N)$, The following conditions must be met:

$$\begin{matrix} V_{6}\equiv\{\{L_{2A}(x=D,\hat{y}_{n}=T,\hat{z}_{n}=N)\leq L_{2A}(x=D,\hat{y}_{n}=P,\hat{z}_{n}=S)\cap(F_{6}\cap F_{1})\} \\ \text{ }\cup\{L_{2A}(x=D,\hat{y}_{n}=T,\hat{z}_{n}=N)\leq L_{2A}(x=D,\hat{y}_{n}=P,\hat{z}_{n}=K)\cap(F_{6}\cap F_{2})\} \\ \text{ }\cup\{L_{2A}(x=D,\hat{y}_{n}=T,\hat{z}_{n}=N)\leq L_{2A}(x=D,\hat{y}_{n}=P,\hat{z}_{n}=N)\cap(F_{6}\cap F_{5})\} \\ \text{ }\cap\{L_{2A}(x=D,\hat{y}_{n}=T,\hat{z}_{n}=N)\leq L_{2A}(x=D,\hat{y}_{n}=T,\hat{z}_{n}=S)\cap(F_{6}\cap F_{4})\} \\ \text{ }\cup\{L_{2A}(x=D,\hat{y}_{n}=T,\hat{z}_{n}=N)\leq L_{2A}(x=D,\hat{y}_{n}=T,\hat{z}_{n}=K)\cap(F_{6}\cap F_{5})\} \\ \text{ }\cap\{L_{2A}(x=D,\hat{y}_{n}=T,\hat{z}_{n}=N)\leq L_{2A}(x=VD,\hat{y}_{n}=P,\hat{z}_{n}=Q)\cap(F_{6}\cap F_{7})\} \\ \text{ }\cup\{L_{2A}(x=D,\hat{y}_{n}=T,\hat{z}_{n}=N)\leq L_{2A}(x=VD,\hat{y}_{n}=T,\hat{z}_{n}=Q)\cap(F_{6}\cap F_{8})\}\} \end{matrix}$$

For $(x^{*},y^{*},z^{*})=(VD,P,Q)$, The following conditions must be met:

$$\begin{matrix} V_{7}\equiv\{\{L_{2A}(x=VD,\hat{y}_{n}=P,\hat{z}_{n}=Q)\leq L_{2A}(x=D,\hat{y}_{n}=P,\hat{z}_{n}=S)\cap(F_{7}\cap F_{1})\} \\ \text{ }\cup\{L_{2A}(x=VD,\hat{y}_{n}=P,\hat{z}_{n}=Q)\leq L_{2A}(x=D,\hat{y}_{n}=P,\hat{z}_{n}=K)\cap(F_{7}\cap F_{2})\} \\ \text{ }\cup\{L_{2A}(x=VD,\hat{y}_{n}=P,\hat{z}_{n}=Q)\leq L_{2A}(x=D,\hat{y}_{n}=P,\hat{z}_{n}=N)\cap(F_{7}\cap F_{3})\} \\ \text{ }\cap\{L_{2A}(x=VD,\hat{y}_{n}=P,\hat{z}_{n}=Q)\leq L_{2A}(x=D,\hat{y}_{n}=T,\hat{z}_{n}=S)\cap(F_{7}\cap F_{4})\} \\ \text{ }\cup\{L_{2A}(x=VD,\hat{y}_{n}=P,\hat{z}_{n}=Q)\leq L_{2A}(x=D,\hat{y}_{n}=T,\hat{z}_{n}=K)\cap(F_{7}\cap F_{5})\} \\ \text{ }\cup\{L_{2A}(x=VD,\hat{y}_{n}=P,\hat{z}_{n}=Q)\leq L_{2A}(x=D,\hat{y}_{n}=T,\hat{z}_{n}=N)\cap(F_{7}\cap F_{6})\} \\ \text{ }\cap\{L_{2A}(x=VD,\hat{y}_{n}=P,\hat{z}_{n}=Q)\leq L_{2A}(x=VD,\hat{y}_{n}=T,\hat{z}_{n}=Q)\cap(F_{7}\cap F_{8})\}\} \end{matrix}$$

For $(x^{*},y^{*},z^{*})=(VD,T,Q)$, The following conditions must be met:

$$\begin{matrix} V_{8}\equiv\{\{L_{2A}(x=VD,\hat{y}_{n}=T,\hat{z}_{n}=Q)\leq L_{2A}(x=D,\hat{y}_{n}=P,\hat{z}_{n}=S)\cap(F_{8}\cap F_{1})\} \\ \text{ }\cup\{L_{2A}(x=VD,\hat{y}_{n}=T,\hat{z}_{n}=Q)\leq L_{2A}(x=D,\hat{y}_{n}=P,\hat{z}_{n}=K)\cap(F_{8}\cap F_{2})\} \\ \text{ }\cup\{L_{2A}(x=VD,\hat{y}_{n}=T,\hat{z}_{n}=Q)\leq L_{2A}(x=D,\hat{y}_{n}=P,\hat{z}_{n}=N)\cap(F_{8}\cap F_{3})\} \\ \text{ }\cap\{L_{2A}(x=VD,\hat{y}_{n}=T,\hat{z}_{n}=Q)\leq L_{2A}(x=D,\hat{y}_{n}=T,\hat{z}_{n}=S)\cap(F_{8}\cap F_{4})\} \\ \text{ }\cup\{L_{2A}(x=VD,\hat{y}_{n}=T,\hat{z}_{n}=Q)\leq L_{2A}(x=D,\hat{y}_{n}=T,\hat{z}_{n}=K)\cap(F_{8}\cap F_{5})\} \\ \text{ }\cup\{L_{2A}(x=VD,\hat{y}_{n}=T,\hat{z}_{n}=Q)\leq L_{2A}(x=D,\hat{y}_{n}=T,\hat{z}_{n}=N)\cap(F_{8}\cap F_{6})\} \\ \text{ }\cap\{L_{2A}(x=VD,\hat{y}_{n}=T,\hat{z}_{n}=Q)\leq L_{2A}(x=VD,\hat{y}_{n}=P,\hat{z}_{n}=Q)\cap(F_{8}\cap F_{7})\}\} \end{matrix}$$
